# Supplementary material for: Peptide nano-blanket impedes fibroblasts activation and subsequent formation of pre-metastatic niche
Source: Nat Commun. 2022 May 25;13:2906. doi: 10.1038/s41467-022-30634-8 (PMC9132894; doi:10.1038/s41467-022-30634-8)
Supplement: Supplementary file 1 — Supplementary information [file 41467_2022_30634_MOESM1_ESM.pdf]

Supplementary Information

Peptide nano-blanket impedes fibroblasts activation and subsequent formation of pre-metastatic niche

Yi Zhou[1], Peng Ke[1 2], Xiaoyan Bao[1], Honghui Wu[1], Yiyi Xia[1], Zhentao Zhang[1], Haiqing Zhong[1], Qi Dai[1 3], Linjie Wu[1], Tiantian Wang[1], Mengting Lin[1], Yaosheng Li[1], Xinchi Jiang[1], Qiyao Yang[1 3], Yiying Lu[1], Xincheng Zhong[1], Min Han[1 4 5], Jianqing Gao[1 4 5]

[1] Institute of Pharmaceutics, Zhejiang Province Key Laboratory of Anti-Cancer Drug Research, College of Pharmaceutical Sciences, Zhejiang University, Hangzhou 310058, PR China.

[2] Shengli Clinical Medical College of Fujian Medical University, Fuzhou 350001, PR China.

[3] Department of Radiation Oncology, Key Laboratory of Cancer Prevention and Intervention, The Second Affiliated Hospital, College of Medicine, Zhejiang University, Hangzhou 310058, PR China.

[4] Cancer Center of Zhejiang University, Zhejiang University, Hangzhou 310058, PR China.

[5] Hangzhou Institute of Innovative Medicine, Zhejiang University, Hangzhou 310058, PR China.

Correspondence should be addressed to M.H. (email: hanmin@zju.edu.cn) and J.G. (email: gaojianqing@zju.edu.cn).

Contents

Supplementary Figure 1. Mass chromatogram of FR17, sFD17 and FG8 4

Supplementary Figure 2. Tandem mass chromatogram of FR17, sFD17 and FG8............... 5

Supplementary Figure 3. Enzyme cleavage of FR17 and sFD17 to release the self-assembled monomer FG8. 6

Supplementary Figure 4. Self-assembly of FG8 in different pH conditions 7

Supplementary Figure 7. The aggregation-induced emission effect of the in-situ assembly of peptide nano-blanket in the lung *in vivo* 10

Supplementary Figure 8. The aggregation-induced emission effect of the in-situ assembly of peptide nano-blanket in heart, spleen, kidney and liver *in vivo* 11

Supplementary Figure 9. Hydrogen Bonds formed in FFKY or FG8 system as a function of time. 11

Supplementary Figure 10. The pathological process in the lung of MCM-induced PMN model *in vivo* 12

Supplementary Figure 11. Immune cell population analysis during the pathological process in the lung of MCM-induced PMN model *in vivo*.. 13

Supplementary Figure 12. Flow cytometry gating strategy for the analysis of immune cell population in pulmonary PMN. 14

Supplementary Figure 13. MCM-induced PMN formation aggravated metastasis *in vivo*. 15

Supplementary Figure 14. The peptide nano-blanket inhibits the activation of lung fibroblasts when induced by MCM 15

Supplementary Figure 15. FR17 administration inhibited extracellular matrix remodeling in pulmonary PMN 16

Supplementary Figure 16. FR17 protected fibroblasts from being irritated by MCM to prevent the disrupting endothelial cell-cell connection and cell proliferation..................... 17

Supplementary Figure 20. The alleviation of PMN development by FR17 administration could be correlated to the cell pathway of myeloid leukocyte migration and the activation of immune response.. 21

Supplementary Figure 21. Effect of FR17 treatment on the proliferation of tumor cells and MLF, and on tumor cell’s migration. 22

Supplementary Figure 25. HPLC of FR17 (%Purity = 98.70). 26

Supplementary Figure 26. HPLC of sFD17 (%Purity = 98.33). 26

Supplementary Figure 27. HPLC of FG8 (%Purity = 98.15). 27

Supplementary Figure 28. Mass chromatogram of TPE-FR17 (MW. 2390.78). 28

Supplementary Figure 29. Mass chromatogram of TPE-FG8 (MW. 1343.59). 28

Supplementary Figure 30. HPLC of TPE-FR17 (%Purity = 98.73). 29

Supplementary Figure 31. HPLC of TPE-FG8 (%Purity = 99.24)...................................... 29

Supplementary Figure 32. The uncropped scans of all blots and gels in Supplementary Figure 10a and Supplementary Figure 15d... 30

Supplementary Table 1. The primer sequences for RT-qPCR analysis. 31

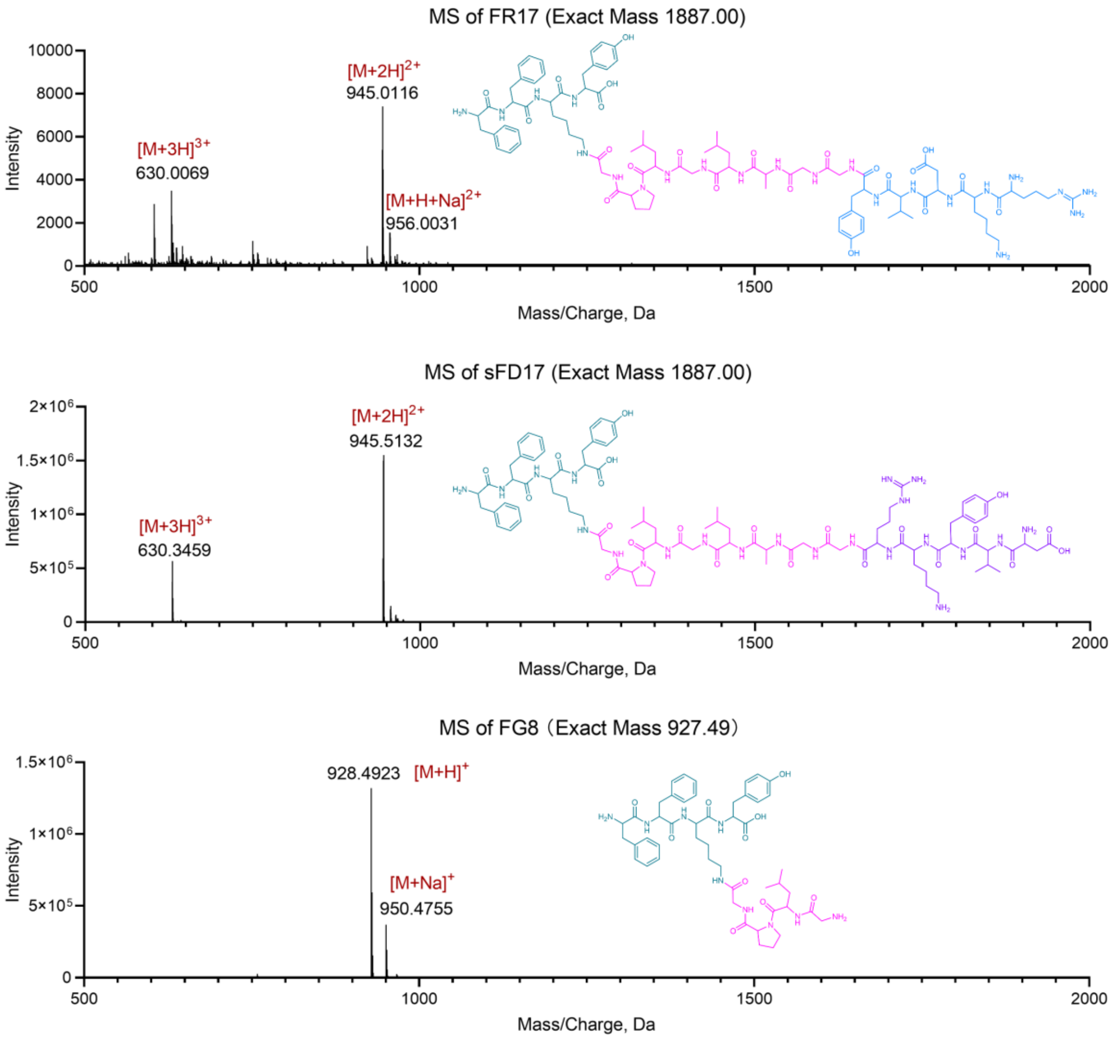

Supplementary Figure 1. Mass chromatogram of FR17, sFD17 and FG8. Mass spectrum and the molecular structure of FR17 (Exact Mass 1887.00), sFD17 (Exact Mass 1887.00) and FG8 (Exact Mass 927.49).

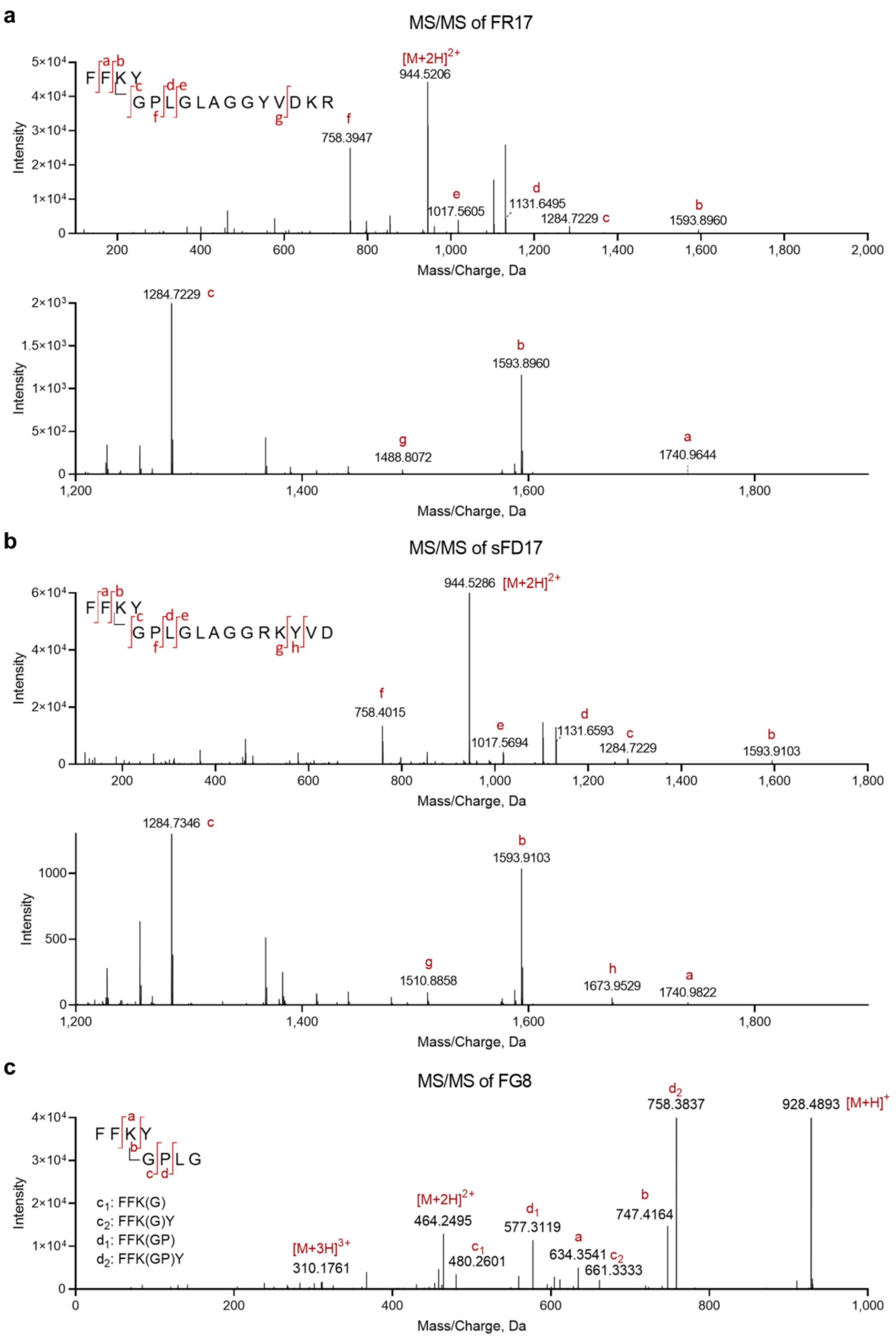

Supplementary Figure 2. Tandem mass chromatogram of FR17, sFD17 and FG8. a, MS/MS of FR17. The red characters a-g represent for different peptide fragments labeled on the top left corner. **b**, MS/MS of sFD17. The red characters a-h represent for different peptide fragments labeled on the top left corner. **c**, MS/MS of FG8. The red characters a-d represent for different peptide fragments labeled on the top left corner.

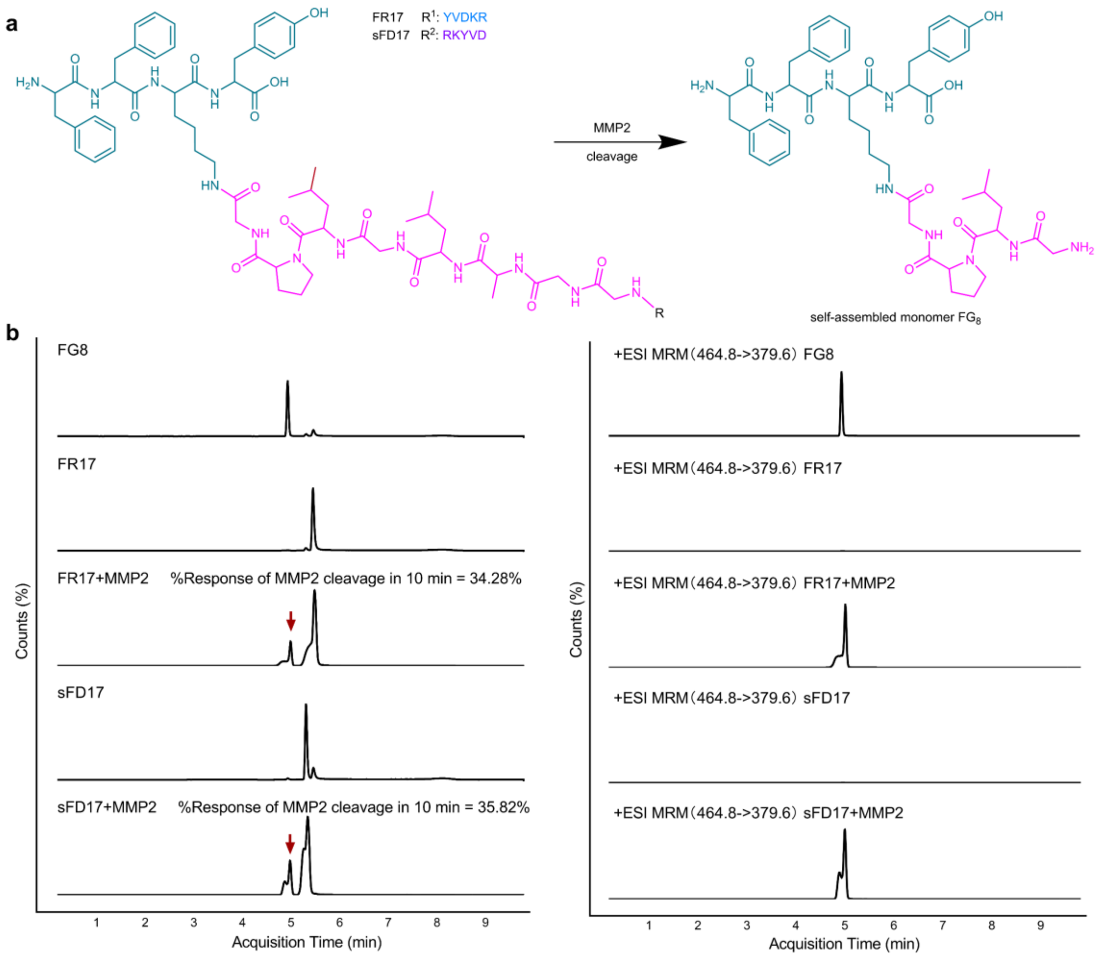

Supplementary Figure 3. Enzyme cleavage of FR17 and sFD17 to release the self-assembled monomer FG8. a, Schematic of the enzyme cleavage of FR17 or sFD17. **b**, Liquid chromatography-tandem mass spectrometry (LC-MS/MS) of FR17 and sFD17 (200 µM) before and after MMP2 (200 ng/ml) treatment. The characteristic peak of FG8 was acquired under the auto-optimized condition of the ion pair 464.8/379.6, in which 464.8 represents $[M+2H]^{2+}$ of FG8 (Exact mass 927.49) and 379.6 represents $[M+2H]^{2+}$ of FFK(GP)Y (Exact mass 757.38).

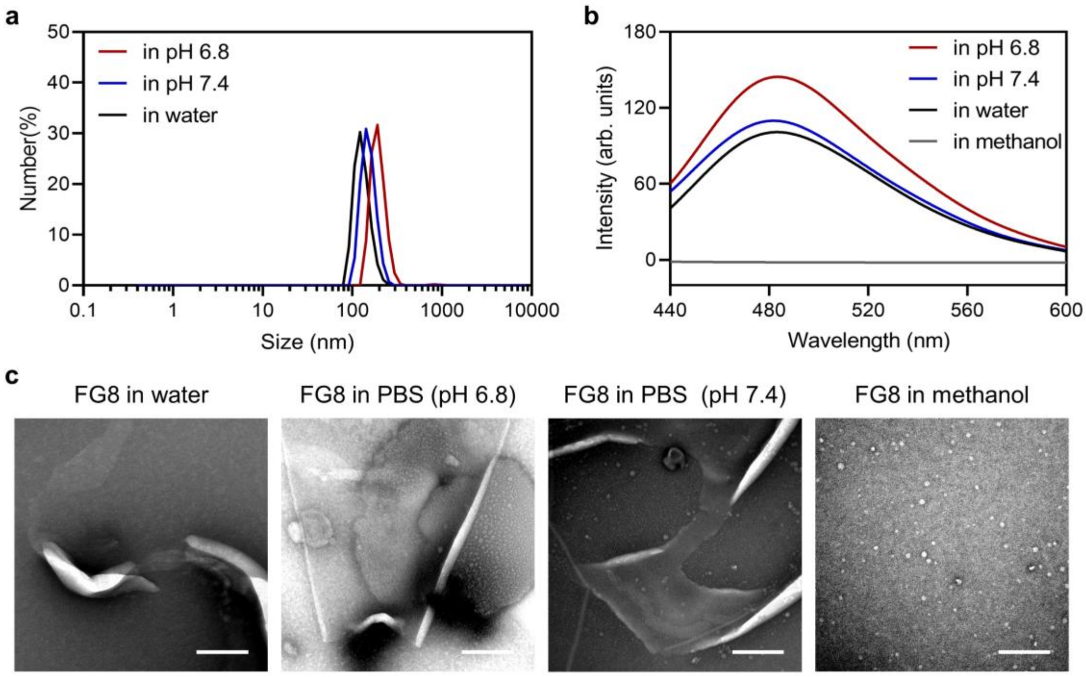

Supplementary Figure 4. Self-assembly of FG8 in different pH conditions. a, Size distribution of the peptide assemblies of FG8 formed in water or PBS (pH 6.8 or pH 7.4). **b**, Fluorescence spectra of the TPE-FG8 (100 μM) in water, PBS (pH 6.8 or pH 7.4) or methanol (dissolved) excited by 405 nm. **c**, TEM images of FG8 assemblies in water, PBS (pH 6.8 or pH 7.4) or methanol (dissolved). Scale bar = 200 nm. Source data are provided as a Source Data file.

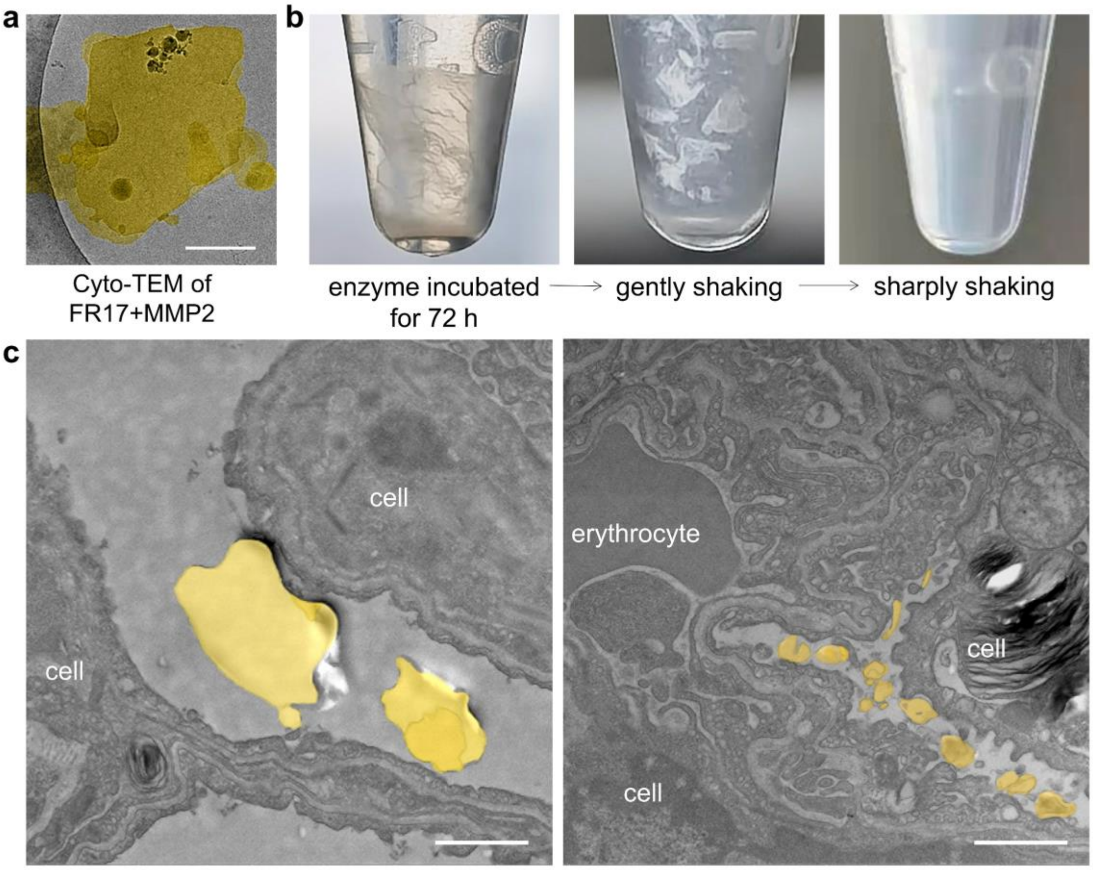

Supplementary Figure 5. Enzyme-activated assembly of FR17 and sFD17. a, Cryo-TEM image of the peptide nano-blanket assembled by FR17 (500 μM) treated with MMP2 (1 μg/ml) for 24 h. Scale bar = 200 nm. **b**, Macroscopic images of the thin layer formed by FR17 treated with enzyme and let stand for 72 h. The soft thin layer broke into pieces after gently shaking and dispersed into nanoscale fragments after sharply shaking. **c**, STEM images of the peptide nano-blanket in the intercellular substance in PMN lung, which was collected from PMN mouse at 12 h-post subcutaneous administration of FR17. Scale bar = 1 μm. The lamellar structure of the peptide nano-blanket was pseudo-colored in gold.

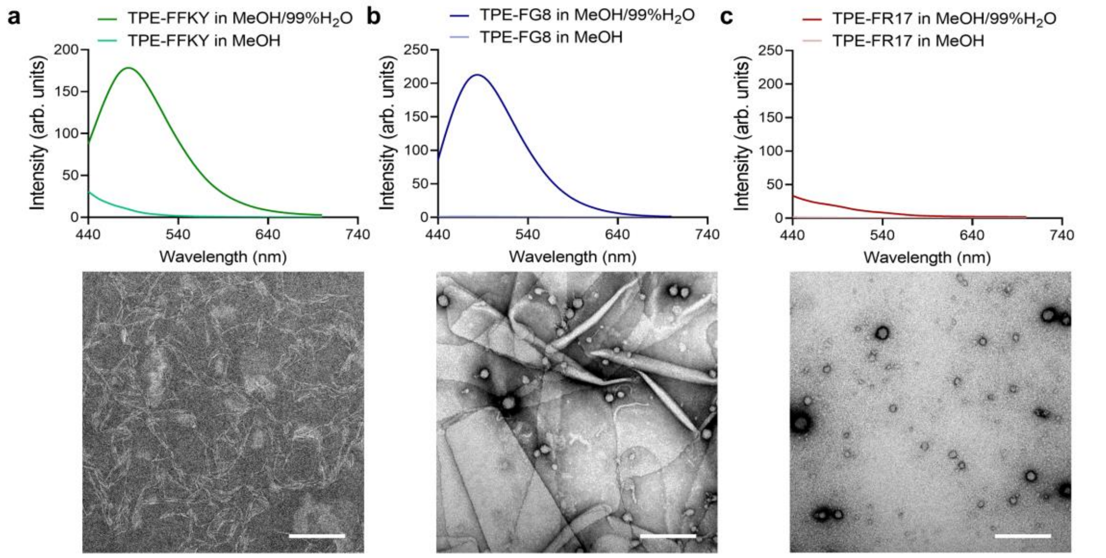

Supplementary Figure 6. The aggregation-induced emission (AIE) effect of TPE-FFKY, TPE-FG8 and TPE-FR17. a, Fluorescence spectra of the TPE-FFKY in methanol (abbreviated as MeOH) (dissolved) and water at methanol fraction of 1% (assembled as illustrated in the TEM image below) excited by 405 nm. Scale bar = 200 nm. **b,** Fluorescence spectra of the TPE-FG8 in methanol (dissolved) and water at methanol fraction of 1% (assembled as illustrated in the TEM image below) excited by 405 nm. Scale bar = 200 nm. **c,** Fluorescence spectra of the TPE-FR17 in methanol and water at methanol fraction of 1% (dispersed as illustrated in the TEM image below). Scale bar = 200 nm. Source data are provided as a Source Data file. Image was representative for 3 independent experiments with similar results.

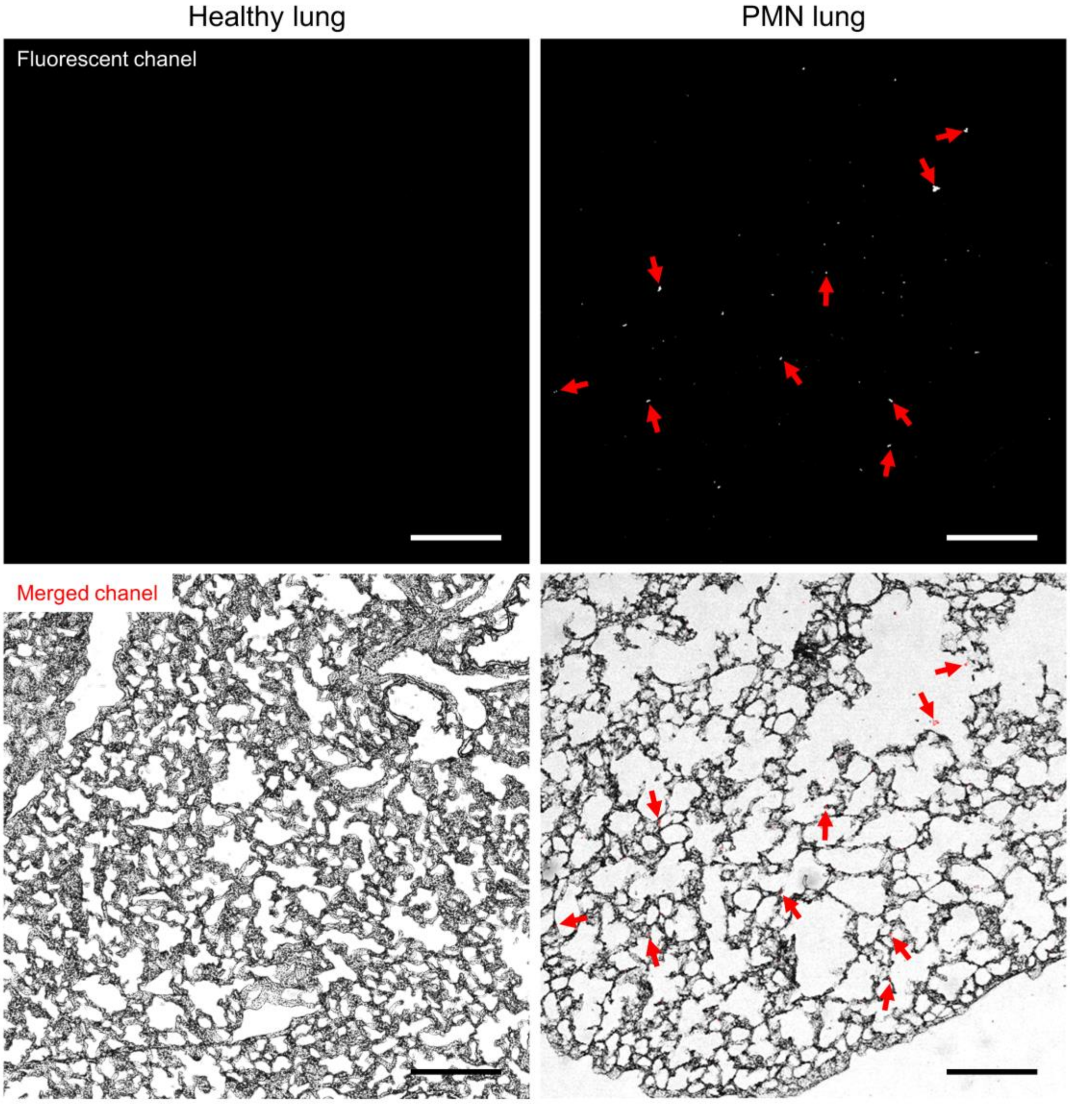

Supplementary Figure 7. The aggregation-induced emission effect of the in-situ assembly of peptide nano-blanket in the lung *in vivo*. Lung secessions at 12 h-post subcutaneous administration of TPE-FR17 (100 μM/kg) in healthy or PMN lung. Peptide assemblies of the monomer TPE-FG8 released from TPE-FR17 in PMN were pseudo-colored in white on the upper panel and pseudo-colored in red in the merged images, and indicated by red arrows for contrast. Scale bar = 200 μm.

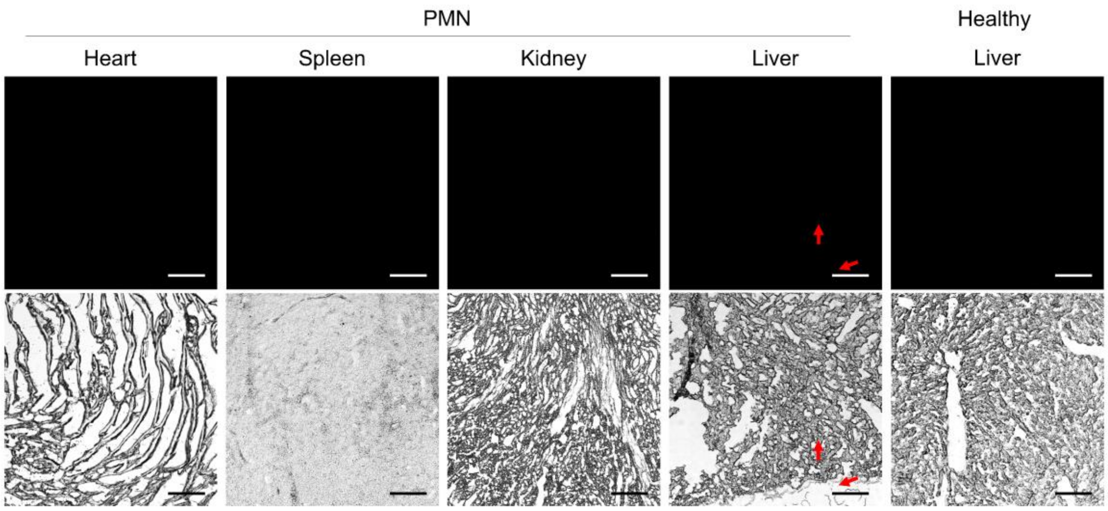

Supplementary Figure 8. The aggregation-induced emission effect of the in-situ assembly of peptide nano-blanket in heart, spleen, kidney and liver *in vivo*. Organs were collected at 12 h-post subcutaneous administration of TPE-FR17 (100 μM/kg) to healthy or PMN mice. Peptide assemblies of the monomer TPE-FG8 released from TPE-FR17 in PMN were pseudo-colored in white on the upper panel and pseudo-colored in red in the merged images, and indicated by red arrows for contrast. Scale bar = 200 μm.

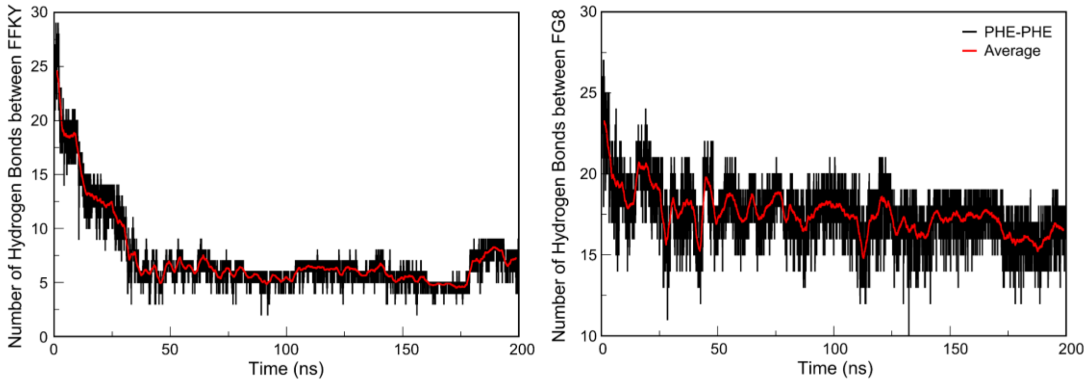

Supplementary Figure 9. Hydrogen Bonds formed in FFKY or FG8 system as a function of time.

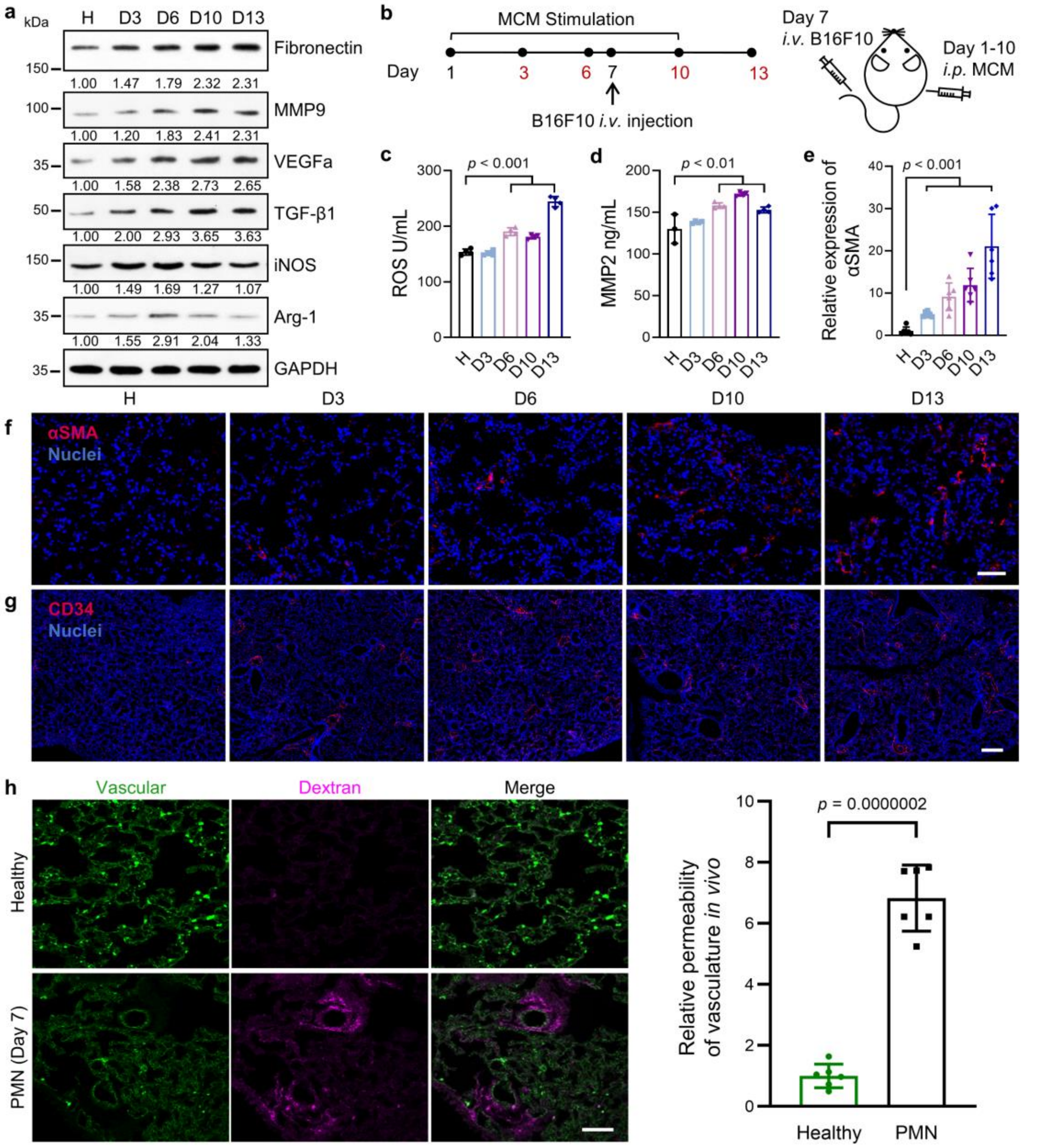

Supplementary Figure 10. The pathological process in the lung of MCM-induced PMN model *in vivo*. **a**, The alteration of Fibronectin, MMP9, VEGFa, TGF-β1, iNOS and Arg-1 expression level in the lung during the development of PMN. **b**, The timeline of MCM-induced PMN model. **c & d**, ROS and MMP2 level in the lung during the development of PMN. Data is presented as mean ± SD. n = 4. One-way ANOVA followed by Tukey's multiple comparisons test was performed. For ROS: H vs. D6, $p = 0.000005$; H vs. D10, $p = 0.000146$; H vs. D13, $p = 0.000000000022$. For MMP2: H vs. D6, $p = 0.0011$; H vs. D10, $p = 0.000018$; H vs. D13, $p = 0.0067$. **e & f,** Representative immunofluorescence images of the lung during the PMN development showing activation of fibroblasts indicated by αSMA^+ labeling. Scale bar = 50 µm. Semi-quantification was gained from six random fields *via* ImageJ. Two-tail *t*-test was performed for statistical evaluation compared to Healthy group. H vs. D3, $p = 0.000007$; H vs. D6, $p = 0.0002$; H vs. D10, $p = 0.000067$; H vs. D13, $p = 0.000073$. **g**, Representative immunofluorescence images of the lung during the PMN development showing angiogenesis indicated by $CD34^+$ labeling. Scale bar = 250 µm. "H", "D3", "D6", "D10", "D13" are the abbreviations of "Healthy", "Day 3", "Day 6", "Day 10", "Day 13" in

the figure labeling. **h**, Vascular permeability of the pulmonary PMN on Day 7. Scale bar = 50 μm. Data is presented as mean ± SD. Semi-quantification was gained from six random fields *via* ImageJ. n = 3 biologically independent mice. Two-tailed unpaired t-test was performed. Source data are provided as a Source Data file.

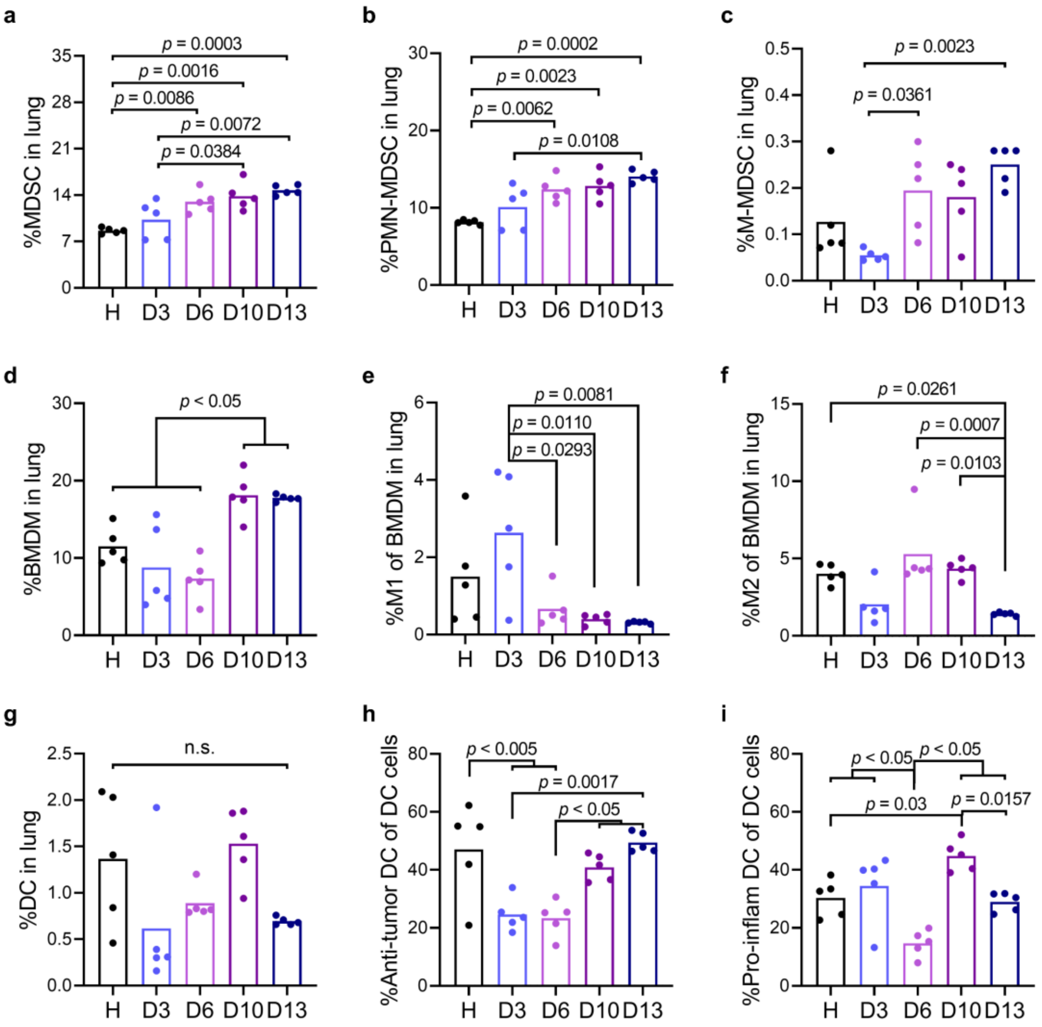

Supplementary Figure 11. Immune cell population analysis during the pathological process in the lung of MCM-induced PMN model *in vivo*. **a**, MDSC recruited to the lung and the alteration of the $CD11b^+Ly6g^+Ly6c^{int}$ cell population PMN-MDSC subtype (**b**) as well as the $CD11b^+Ly6g^-Ly6c^+$ subtype M-MDSC (**c**) during the development of PMN. **d**, BMDM cells recruited to the lung and its sub phenotype M1 (**e**) and M2 (**f**) along with the development of PMN. **g**, Dendritic cell (DC) population in the lung and its sub phenotype $CD103^+$ DC, *i.e.* anti-tumor DC (**h**) and $CD11b^+$ DC, *i.e.* pro-inflam DC (**i**) along with the development of PMN. "H", "D3", "D6", "D10", "D13" are the abbreviations of "Healthy", "Day 3", "Day 6", "Day 10", "Day 13" in the figure labeling. Data is presented as mean ± SD. n = 5 biologically independent mice. One-way ANOVA followed by Tukey's multiple comparisons test was performed for data analysis. For BMDM in lung: H vs. D10,

p = 0.0280; H vs. D13, *p* = 0.0407; D3 vs. D10, *p* = 0.0014; D3 vs. D13, *p* = 0.0021; D6 vs. D10, *p* = 0.0003; D6 vs. D13, *p* = 0.0004. For Anti-tumor DC of DC cells: H vs. D3, *p* = 0.0045; H vs. D6, *p* = 0.0026; D3 vs. D13, *p* = 0.0017; D6 vs. D10, *p* = 0.0314; D6 vs. D13, *p* = 0.0010. For Pro-inflam DC of DC cells: H vs. D6, *p* = 0.0174; H vs. D10, *p* = 0.0300; D3 vs. D6, *p* = 0.0023; D6 vs. D10, *p* = 0.000014; D6 vs. D13, *p* = 0.0332; D10 vs. D13, *p* = 0.0157. Source data are provided as a Source Data file.

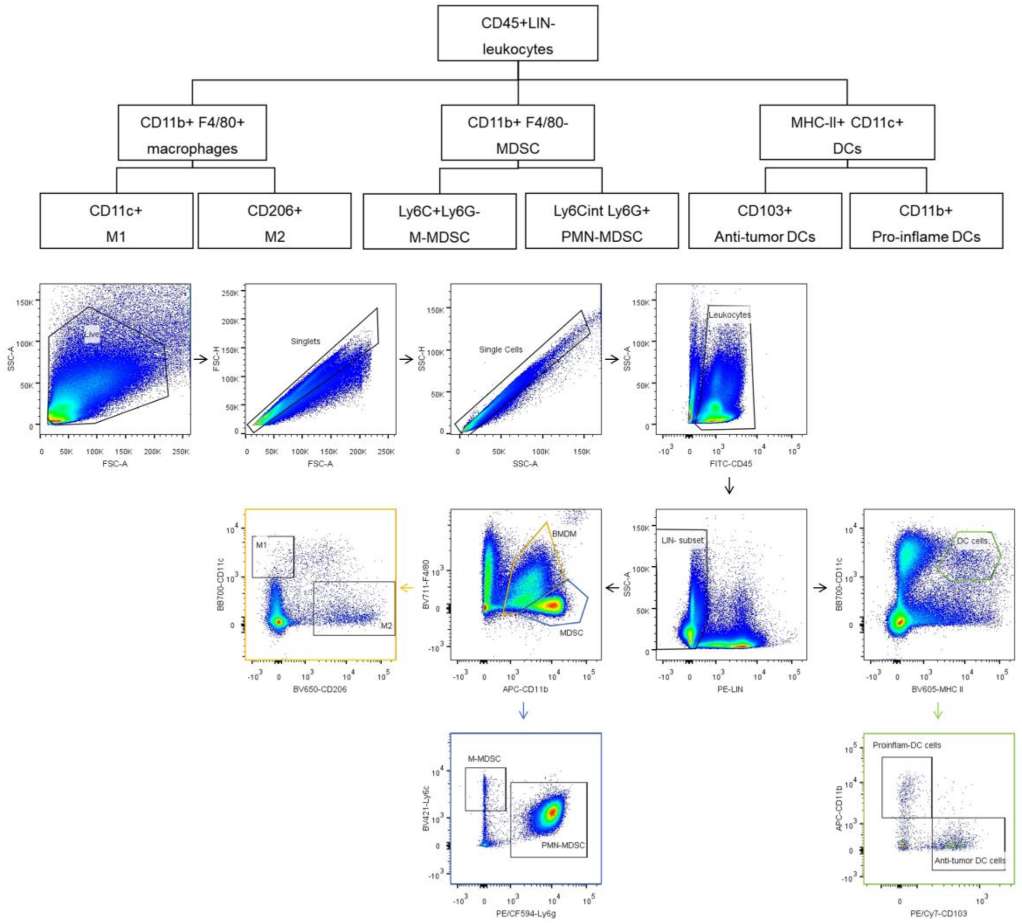

Supplementary Figure 12. Flow cytometry gating strategy for the analysis of immune cell population in pulmonary PMN.

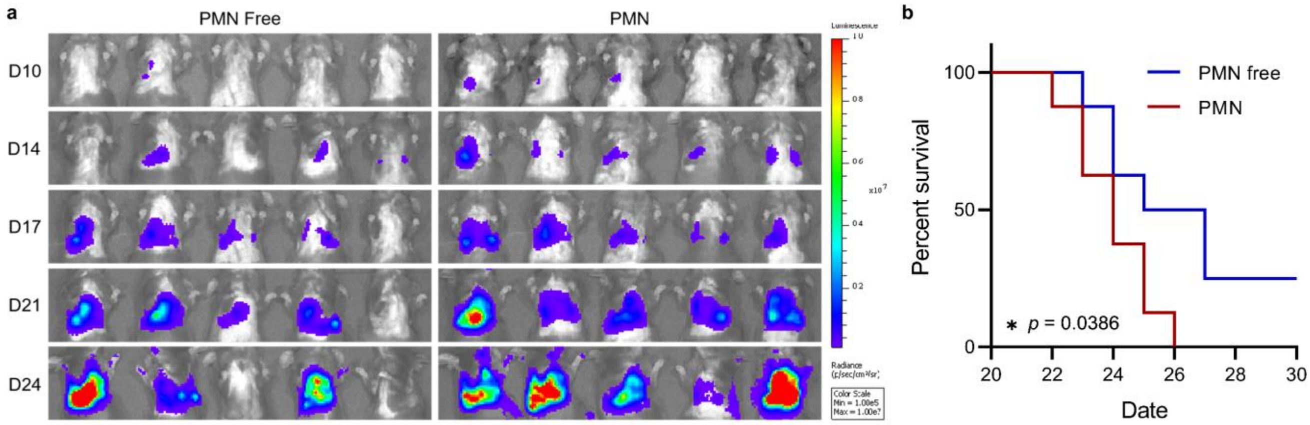

Supplementary Figure 13. MCM-induced PMN formation aggravated metastasis *in vivo*. a, *In vivo* bioluminescent images of the lungs of mice with or without MCM-induced PMN. n = 5. **b**, Survival curves of the lung metastatic mice with or without PMN in 30 days. n = 8. Logrank (Mantel-Cox test) was performed for curve comparison. Source data are provided as a Source Data file.

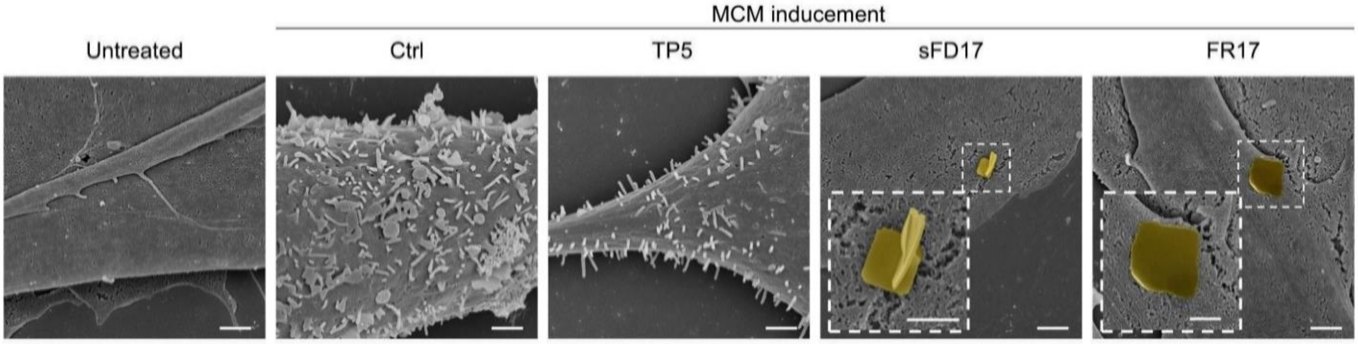

Supplementary Figure 14. The peptide nano-blanket inhibits the activation of lung fibroblasts when induced by MCM. The enhanced microvilli on the cell surface suggest the activation state of fibroblasts aroused by tumor-derived factors contained in MCM, while the smooth surface indicates the resting state of fibroblasts. The specifically assembled peptide nano-blanket on the surface of fibroblasts is pseudo-colored in gold. Scale bar = 1 μm in the macro images. Scale bar = 500 nm in the enlarged images circled with dash line.

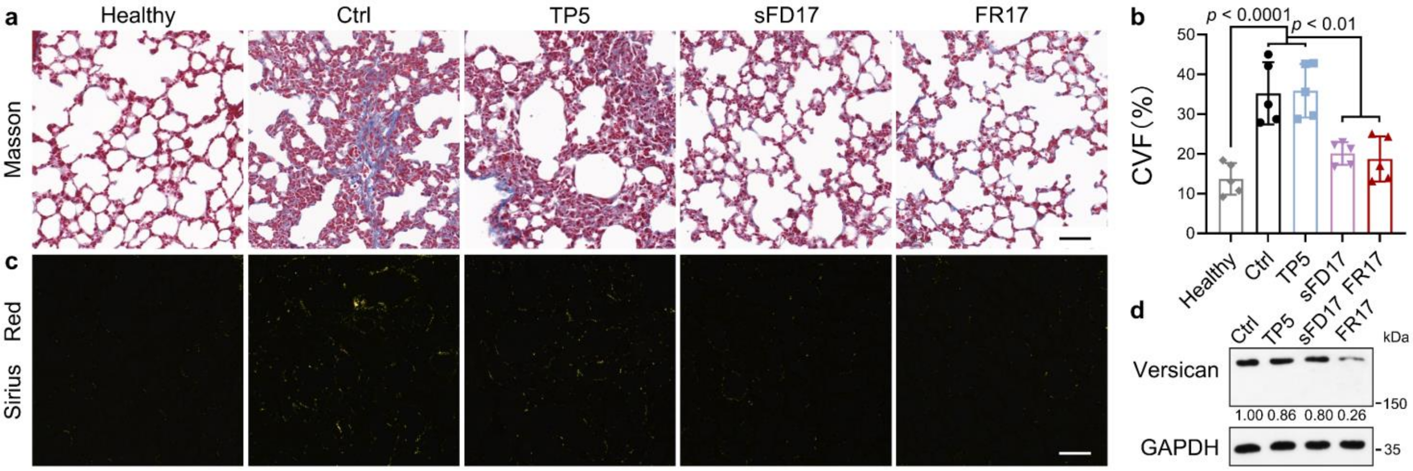

Supplementary Figure 15. FR17 administration inhibited extracellular matrix remodeling in pulmonary PMN. a, b, Representative images of collagen deposition in the lung harvested from the model mice administrated with different peptides by Masson staining. Scale bar = 50 µm. Semi-quantification was gained from five random fields *via* ImageJ. Collagen volume fraction (CVF) was calculated by normalizing the blue collagen area to the total tissue area. Data is presented as mean ± SD. One-way ANOVA followed by Tukey's multiple comparisons test was employed for data analysis. Healthy vs. Ctrl, $p = 0.000071$; Healthy vs. TP5, $p = 0.000048$; Ctrl vs. sFD17, $p = 0.0037$; Ctrl vs. FR17, $p = 0.0016$; TP5 vs. sFD17, $p = 0.0024$; TP5 vs. FR17, $p = 0.0010$. **c**, Representative images of the Sirius Red Staining sections of the lung harvested from the model mice administrated with different peptides on Day 10 taken by polarizing microscope to show the deposited collagen is mainly collagen fiber IV. Scale bar = 50 µm. **d**, Expression of versican in the lung harvested from the PMN model mice administrated with different peptides on Day 10. Source data are provided as a Source Data file.

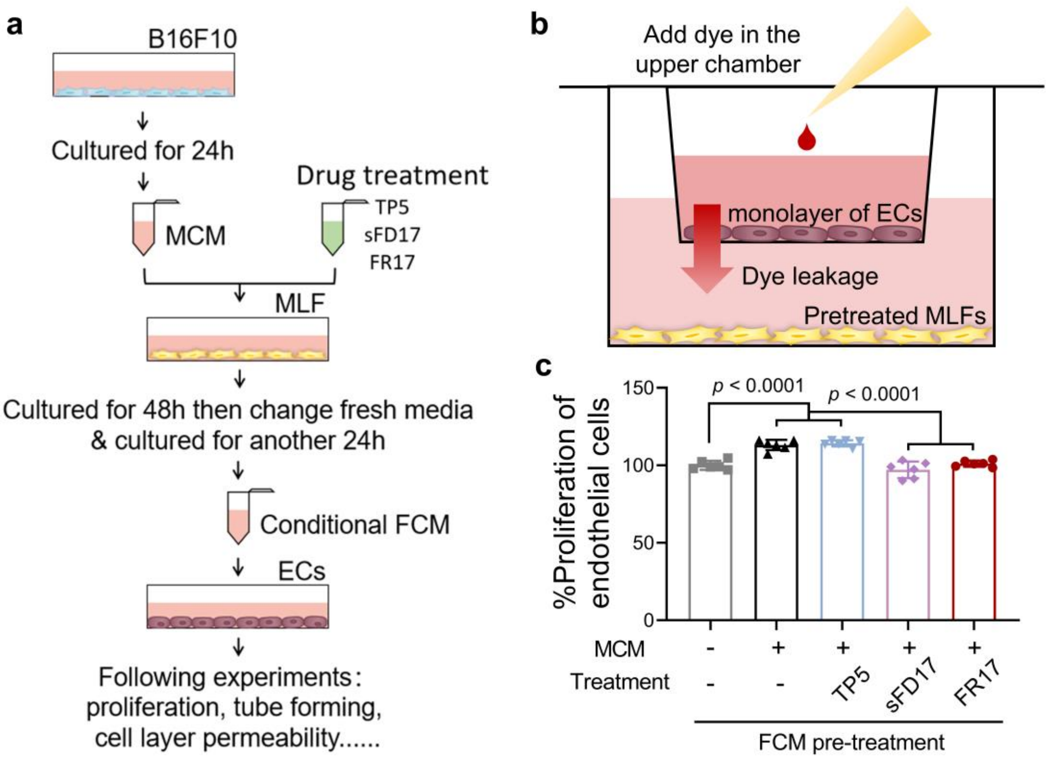

Supplementary Figure 16. FR17 protected fibroblasts from being irritated by MCM to prevent the disrupting endothelial cell-cell connection and cell proliferation. a, The experimental procedure to obtain the conditional FCM after MCM stimulation and the peptide treatment on MLF *in vitro* for further experiments on endothelial cells. **b**, Schematic illustration of the transwell permeability assay. **c**, Proliferation of the endothelial cells cultivated with conditional FCM. Data is presented as mean ± SD. n = 6. One-way ANOVA followed by Tukey's multiple comparisons test was employed for data analysis. FCM vs. MCM, $p = 0.000004$; FCM vs. TP5, $p = 0.000001$; MCM vs. sFD17, $p = 0.00000012$; MCM vs. FR17, $p = 0.000016$; TP5 vs. sFD17, $p = 0.00000007$; TP5 vs. FR17, $p = 0.000004$. Source data are provided as a Source Data file.

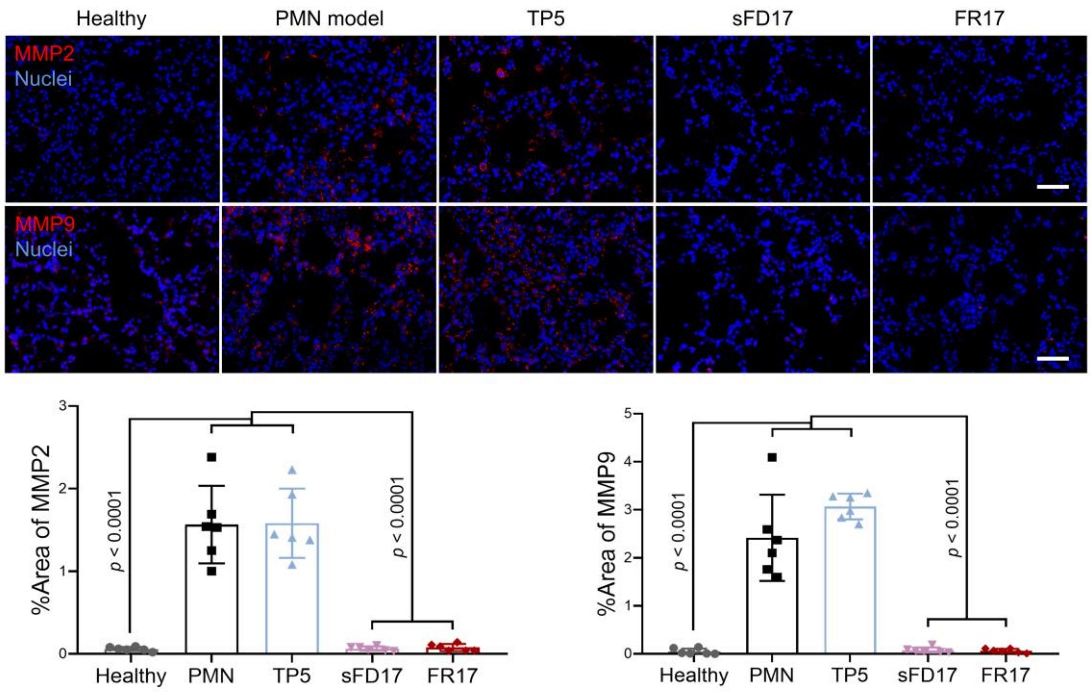

Supplementary Figure 17. FR17 administration down-regulated matrix metalloproteinase in pulmonary PMN. Representative images of MMP2 and MMP9 in the lung harvested from the PMN model mice administrated with different peptides. Scale bar = 50 μm. Semi-quantification was calculated from six random fields *via* ImageJ. Data is presented as mean ± SD. One-way ANOVA followed by Tukey's multiple comparisons test was employed for data analysis. For Area of MMP2: Healthy vs. PMN, $p = 0.00000002$; Healthy vs. TP5, $p = 0.00000001$; PMN vs. sFD17, $p = 0.00000002$; PMN vs. FR17, $p = 0.00000002$; TP5 vs. sFD17, $p = 0.00000001$; TP5 vs. FR17, $p = 0.00000002$. For Area of MMP9: Healthy vs. PMN, $p = 0.0000000052$; Healthy vs. TP5, $p = 0.000000000034$; PMN vs. sFD17, $p = 0.0000000065$; PMN vs. FR17, $p = 0.0000000058$; TP5 vs. sFD17, $p = 0.000000000041$; TP5 vs. FR17, $p = 0.000000000037$. Source data are provided as a Source Data file.

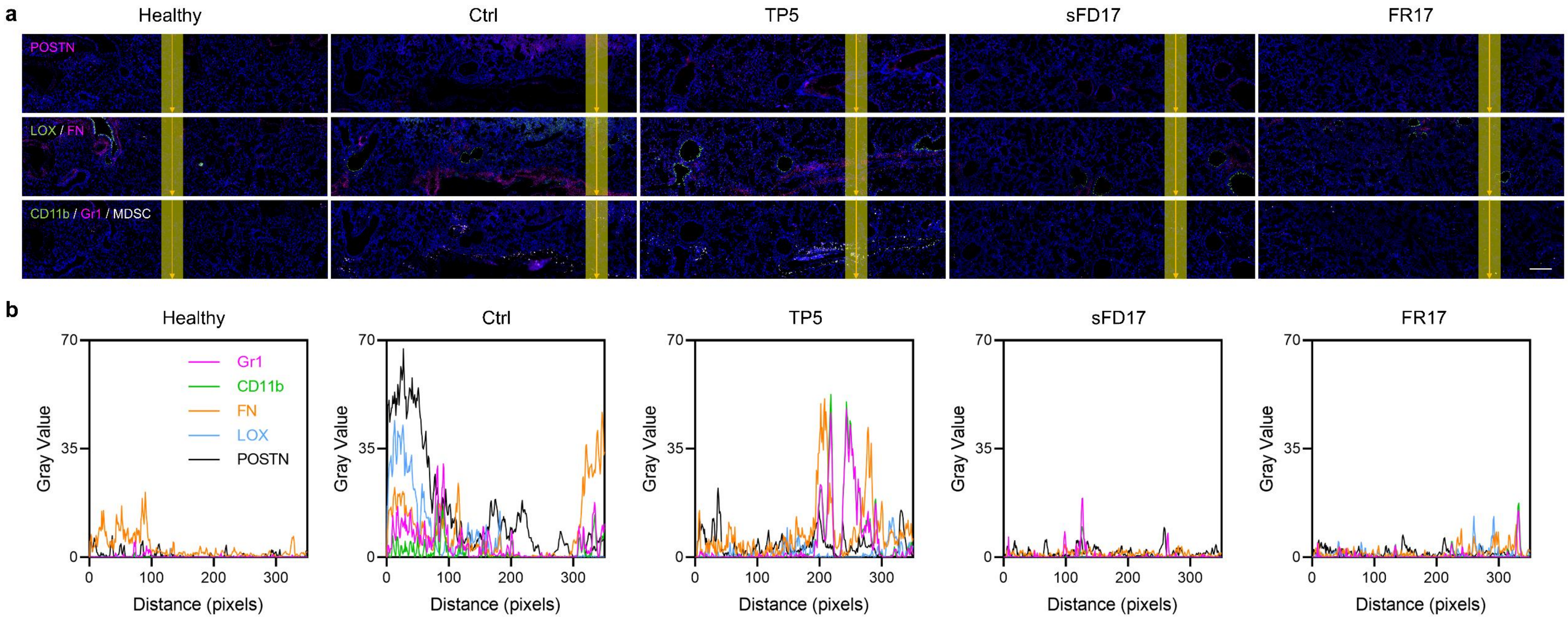

Supplementary Figure 18. FR17 administration prevented MDSC recruitment to pulmonary PMN by influencing the extracellular matrix remodeling. a, Images under low magnification ratio of the serial sections of the lungs harvested from the model mice treated with different peptides were taken. Serial sections show the co-location and distribution of periostin (POSTN, magenta), lysyl oxidase (LOX, green) and Fibronectin (FN, magenta), $CD11b^{+}Gr1^{+}$ MDSC (white merged from green and magenta). Scale bar = 200 µm. **b**, The co-location analysis of $CD11b^{+}Gr1^{+}$ MDSC and POSTN, LOX, FN alongside the yellow arrow marked on the above panel *via* ImageJ. Source data are provided as a Source Data file.

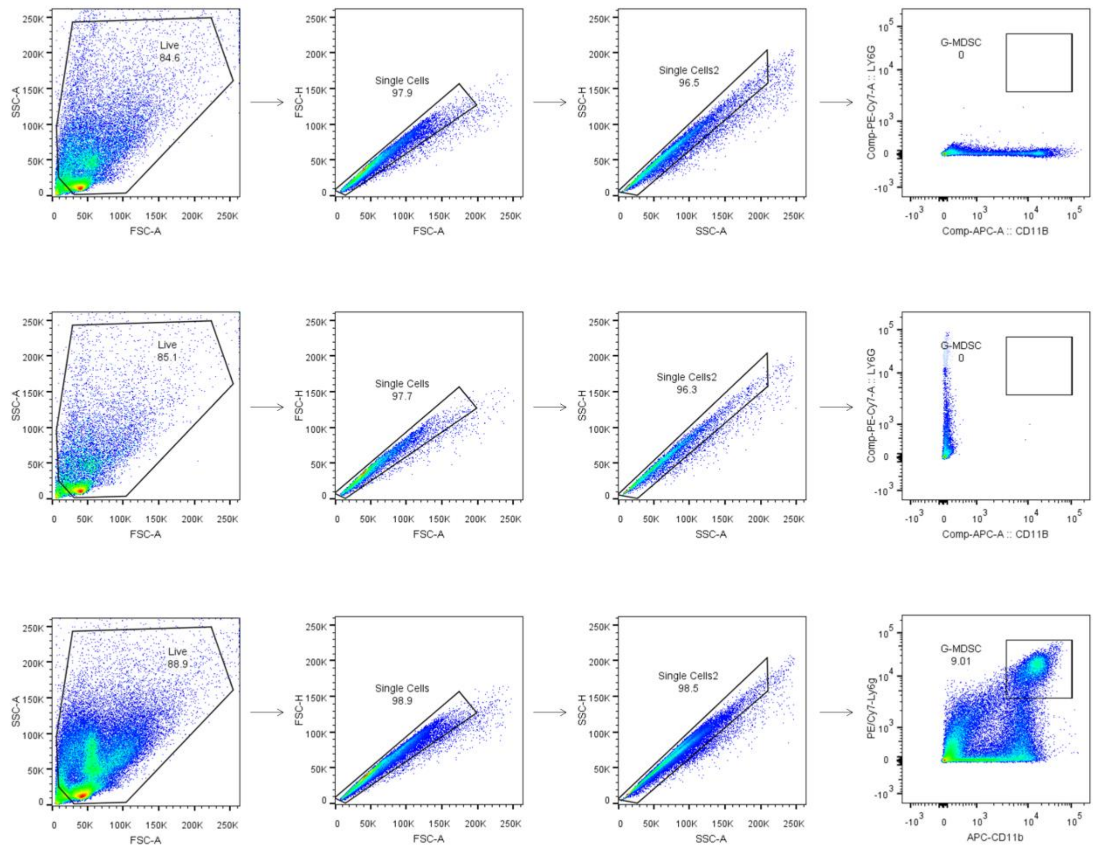

Supplementary Figure 19. Flow cytometry gating strategy for the analysis of CD11b^{+}Ly6g^{+} MDSC recruited to the pulmonary PMN.

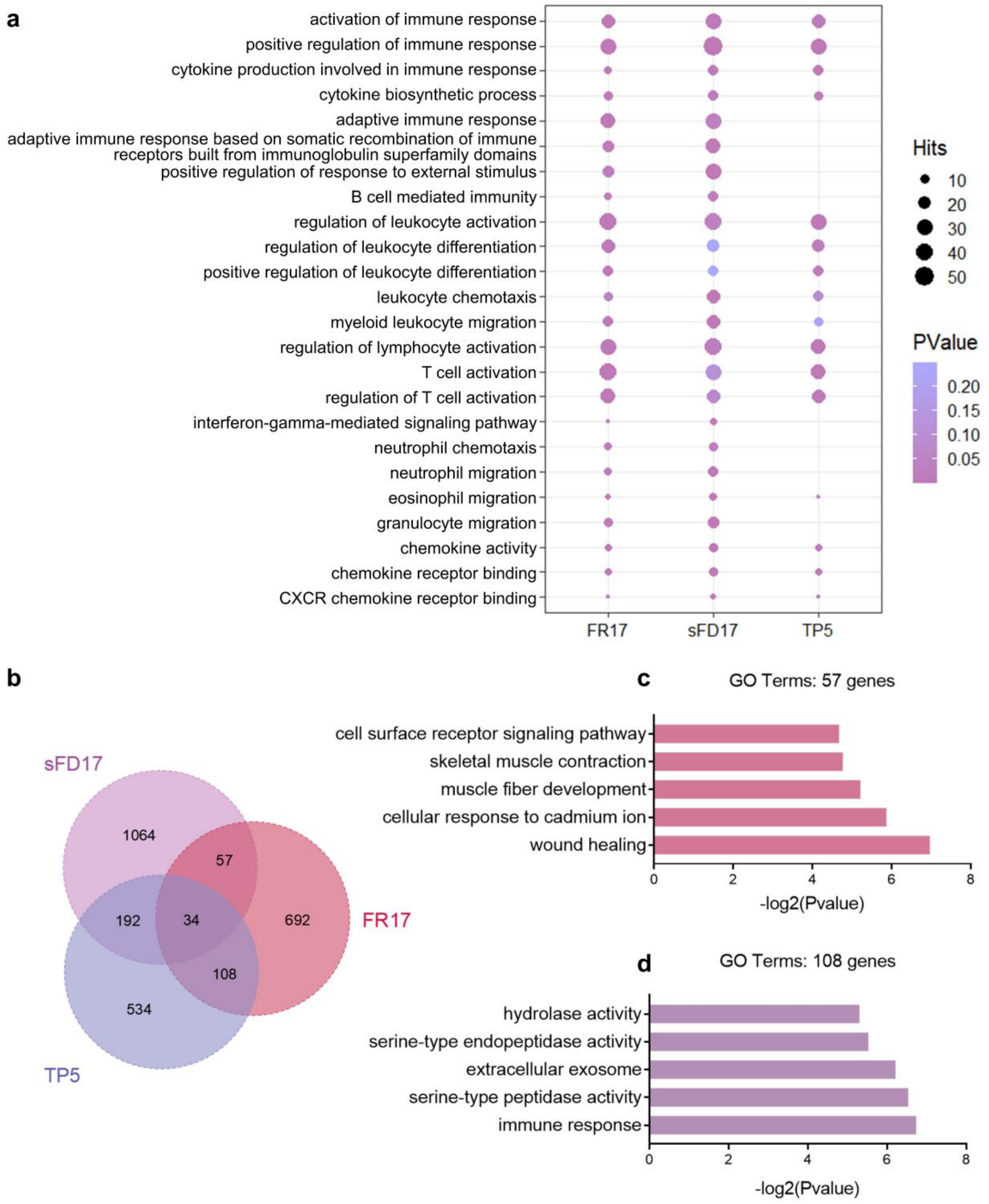

Supplementary Figure 20. The alleviation of PMN development by FR17 administration could be correlated to the cell pathway of myeloid leukocyte migration and the activation of immune response. GO functional analysis was performed in the DAVID database[1], using Fisher's Exact test to compare differently expressed genes and gene backgrounds to measure the gene-enrichment in annotation terms. **a**, GO enrichment analysis of CD11b$^+$Ly6g$^+$ MDSC sorted from different treatment groups on Day 10. RNA preparations were extracted from CD11b$^+$Ly6g$^+$ MDSCs sorted from lungs pooled from 10–12 mice per sample. The bubble plot was drawn by ggplot2 package[2] in R software. The size of the dots corresponds to the number of genes per pathway, and the color indicates *p*-value. **b**, Venn diagram of the data gained from mRNA sequencing of CD11b$^+$Ly6g$^+$

MDSC sorted from different treated mice. Numbers of mRNA whose expressions altered and had significance with the control group are shown in the blue, purple and red regions respectively. *P* values was calculated according to negative binomial distribution. Significance was determined as $p < 0.05$. The diagram was drawn using the VennDiagram package[3]. **c**, GO Enrichment analysis of the 57 mRNA regulated by the peptide nano-blanket (*i.e.*, the overlapping alteration in sFD17 and FR17 treated groups while excluding TP5 effect). **d**, GO Enrichment analysis of the 108 mRNA regulated by the amino acid sequence of TP5 (*i.e.*, the overlapping alteration in TP5 and FR17 treated groups while excluding sFD17 effect). Source data are provided as a Source Data file. The mRNA-seq datasets are available in the NCBI GEO repository, accession code GSE181898.

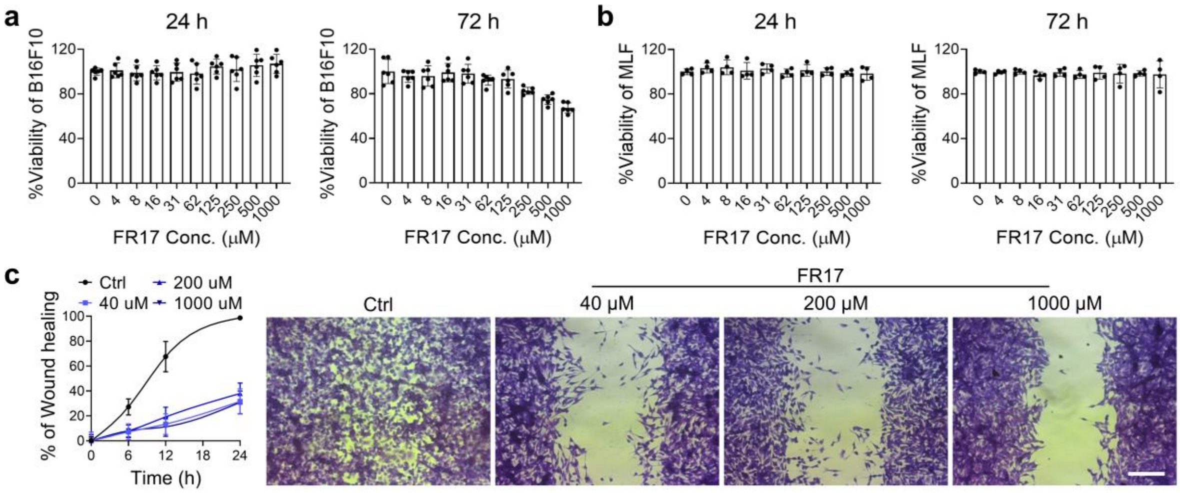

Supplementary Figure 21. Effect of FR17 treatment on the proliferation of tumor cells and MLF, and on tumor cell's migration. **a**, Proliferation of B16F10 treated with FR17 at different concentrations for 24 h or 72 h. Data is presented as mean ± SD. n = 6. **b**, Proliferation of MLF treated with FR17 at different concentrations for 24 h or 72 h. Data is presented as mean ± SD. n = 4. **c**, Migration of B16F10 when treated with FR17 at different concentrations for 24 h. Data is presented as mean ± SD. n = 3. Scale bar = 200 μm. Source data are provided as a Source Data file.

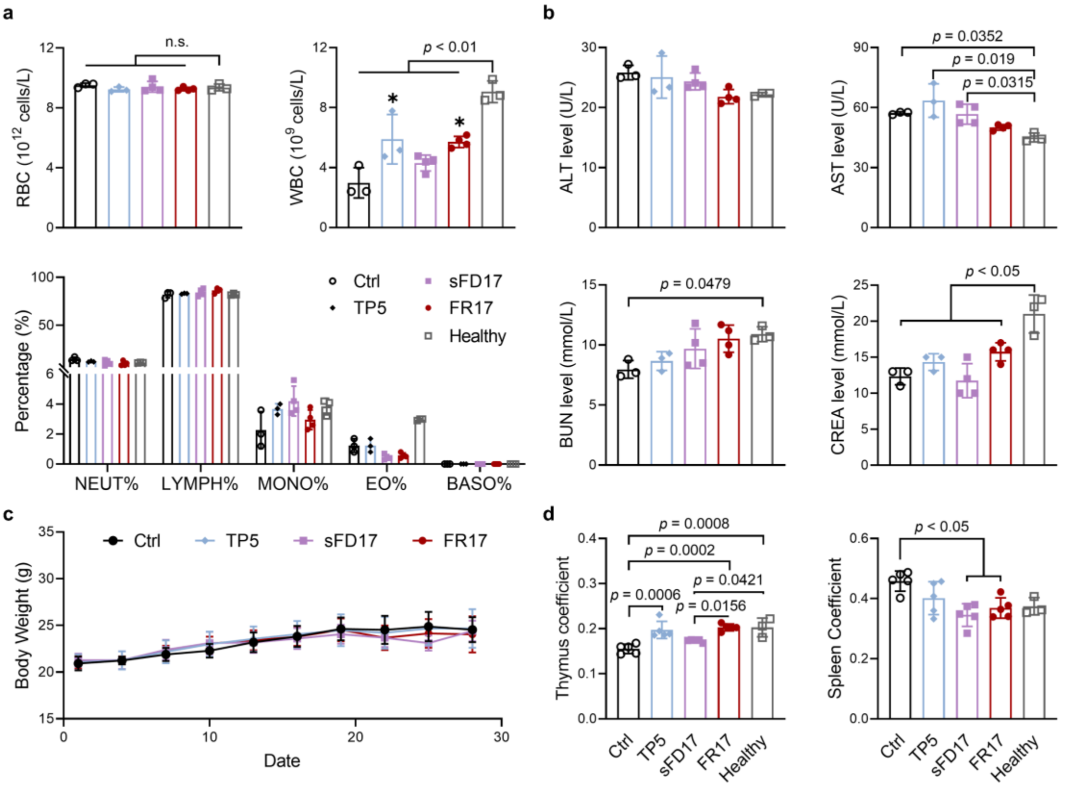

Supplementary Figure 22. Preliminary safety evaluation of peptide administration on lung metastasis model with MCM-induced PMN *in vivo*. a, Complete blood count conducted on the peripheral blood including red blood cell (RBC), white blood cell (WBC), neutrophil (NEUT), lymphocyte (LYMPH), monocyte (MONO), eosinophil (EO), basophil (BASO). For WBC: Ctrl vs. Healthy, p = 0.000023; TP5 vs. Healthy, p = 0.0075; sFD17 vs. Healthy, p = 0.0001; FR17 vs. Healthy, p = 0.0029. **b**, Hepatic and renal function reflected by alanine aminotransferase (ALT), aspartate aminotransferase (AST), blood urea nitrogen (BUN) and serum creatinine (CREA). Blood samples were collected on Day 20 from the lung metastasis model mice administrated with different peptides. Data is presented as mean ± SD. n = 4 biologically independent mice for peptide treated groups and 3 for control and healthy groups. For CREA level: Ctrl vs. Healthy, p = 0.0007; TP5 vs. Healthy, p = 0.0060; sFD17 vs. Healthy, p = 0.0002; FR17 vs. Healthy, p = 0.0199. **c**, Weight curve of the lung metastasis model mice administrated with different peptides. Data is presented as mean ± SD. n = 7. **d**, Thymus coefficient and spleen coefficient of the lung metastasis model mice administrated with different peptides at the end of the experiment. The organ coefficient was calculated by normalizing the organ's weight to the body weight. Data is presented as mean ± SD. n = 5 for peptide treated groups and control group. n = 3 for healthy group. For Spleen Coefficient: Ctrl vs. sFD17, p = 0.0024; Ctrl vs. FR17, p = 0.0167. One-way ANOVA followed by Tukey's multiple comparisons test was employed for data analysis in **a, b** & **d**. Source data are provided as a Source Data file.

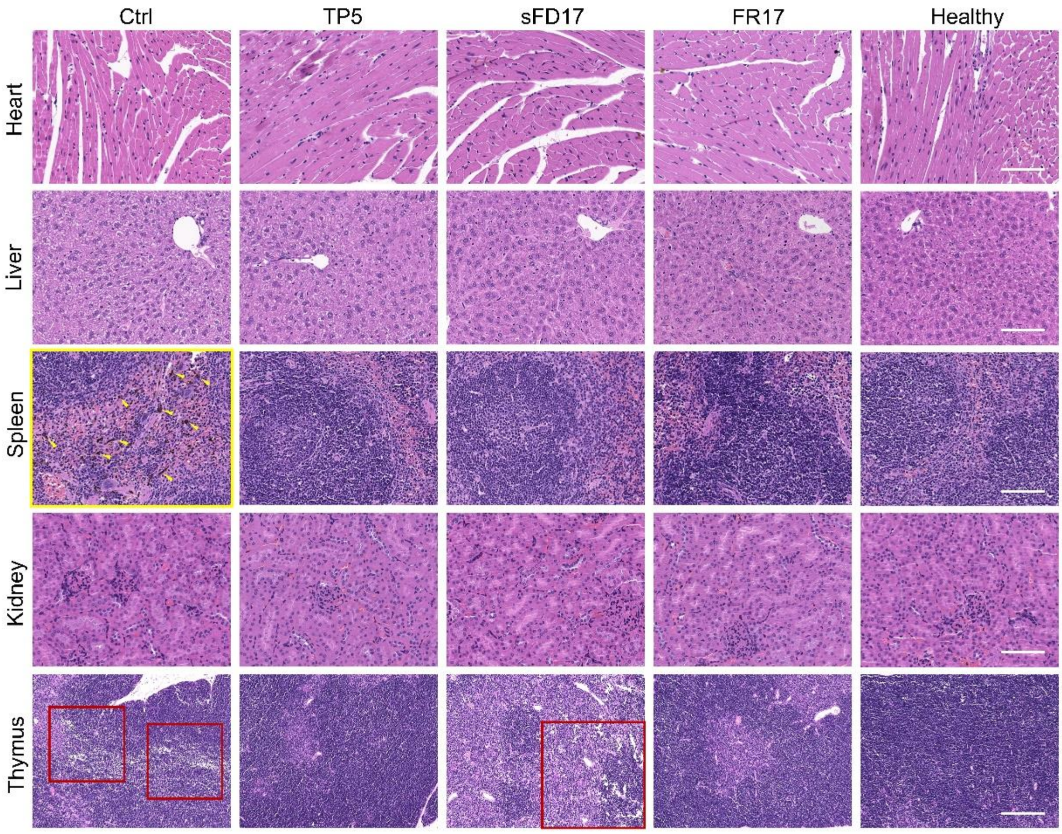

Supplementary Figure 23. Preliminary safety evaluation of peptide administration by the Hematoxylin & Eosin staining of major organs. The yellow arrows indicate the infiltrated B16F10 cells into the spleen of the control mice. The red boxes show slightly atrophied of the thymuses with the unoccupied zones. Scale bar = 100 μm for heart, liver, spleen and kidney sections. Scale bar = 200 μm for thymus sections.

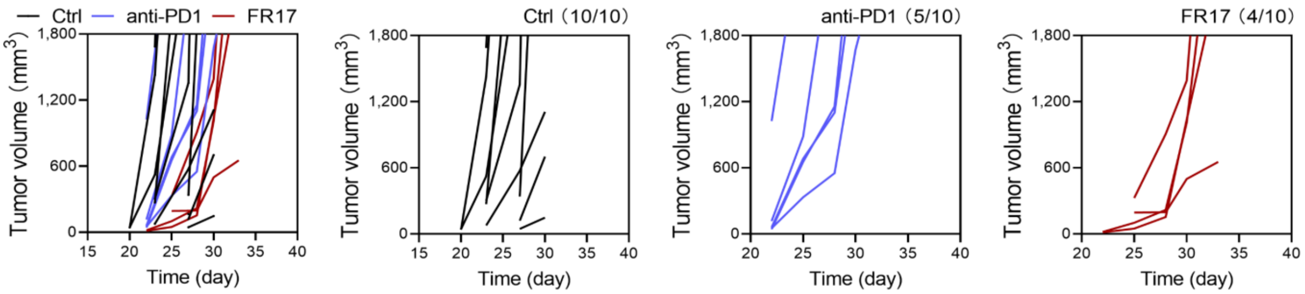

Supplementary Figure 24. Tumor volume curve of the recurrent tumor on the back of mice post-surgery. n = 10 relapses after the surgery in 10 mice for the control group, n = 5 relapses after the surgery in 10 mice for anti-PD1 group, n = 4 relapses after the surgery in 10 mice for FR17 group. Source data are provided as a Source Data file.

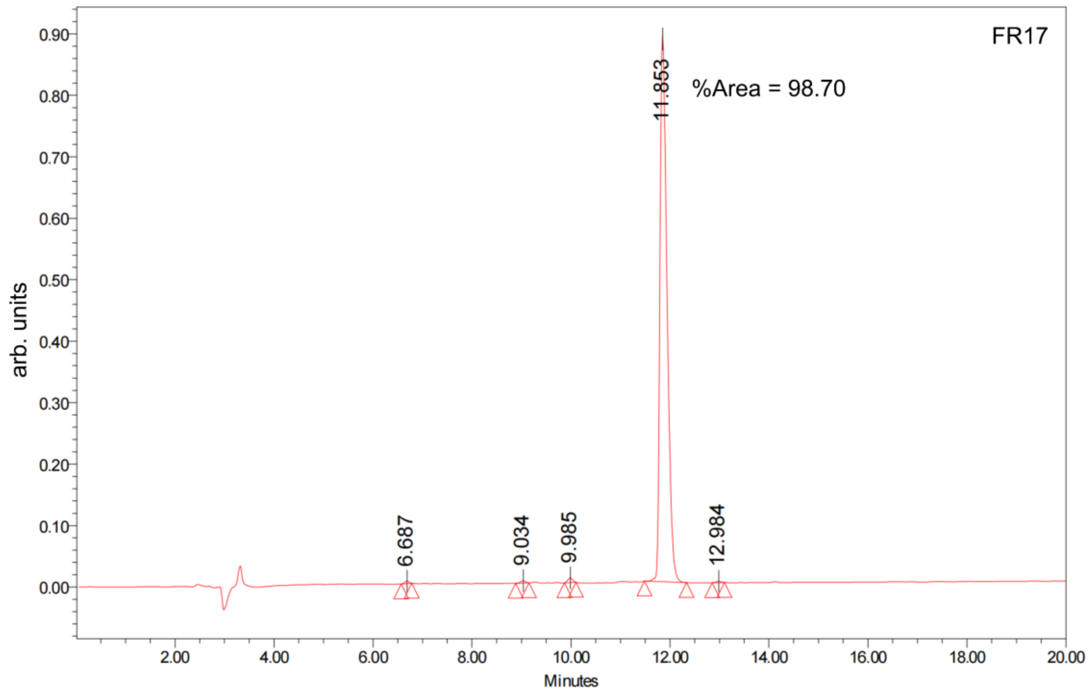

Supplementary Figure 25. HPLC of FR17 (%Purity = 98.70).

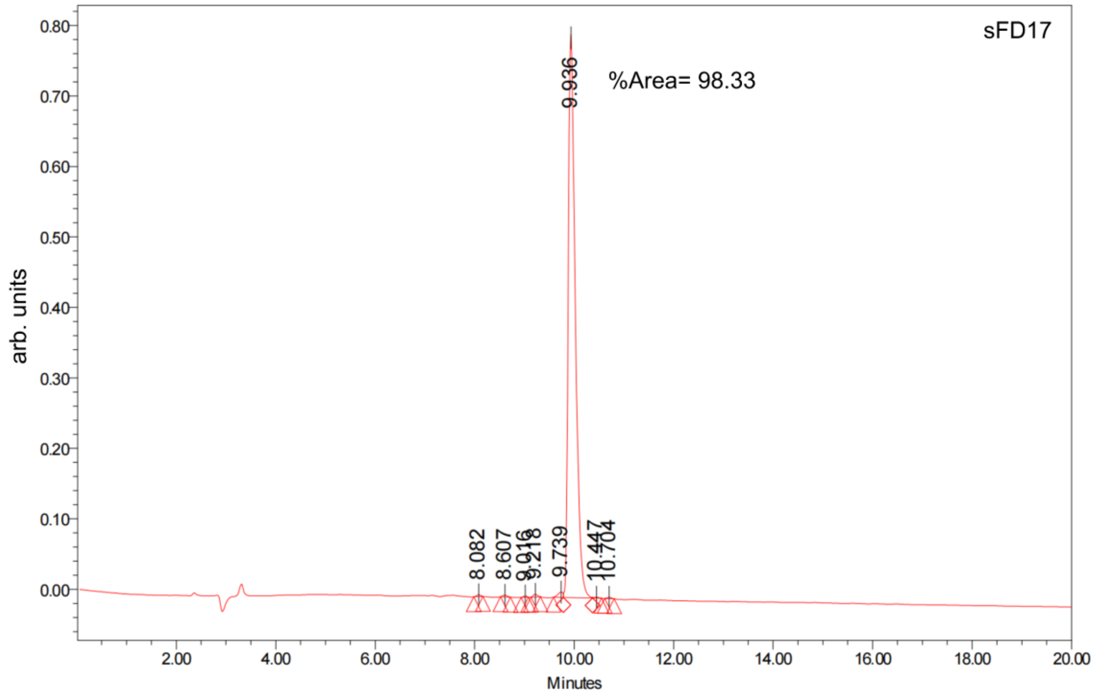

Supplementary Figure 26. HPLC of sFD17 (%Purity = 98.33).

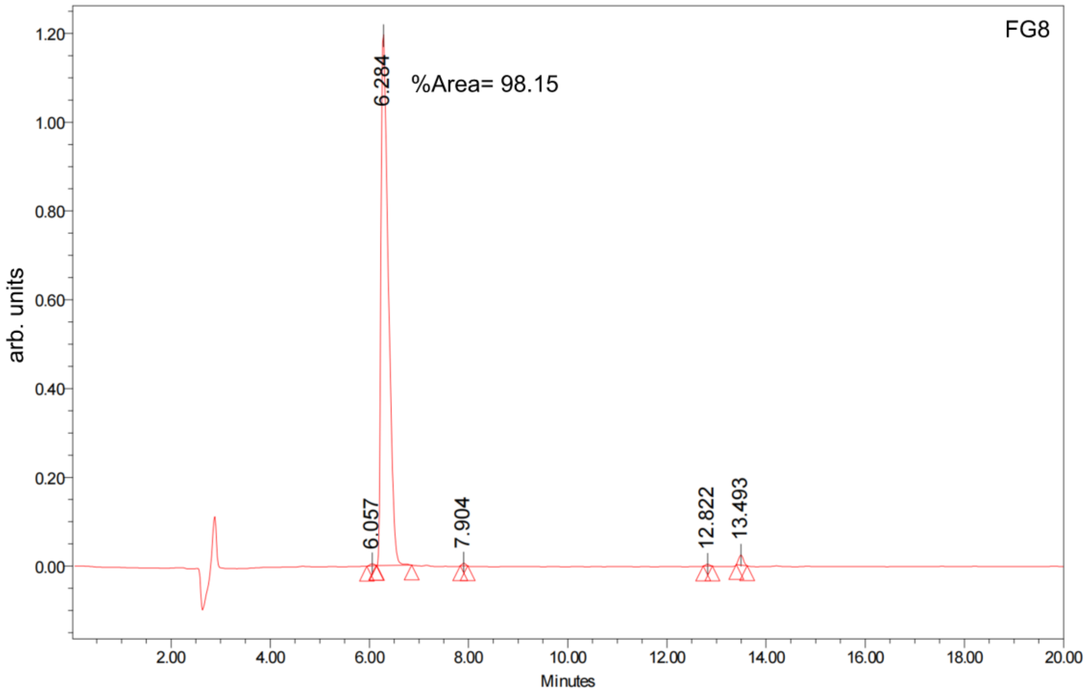

Supplementary Figure 27. HPLC of FG8 (%Purity = 98.15).

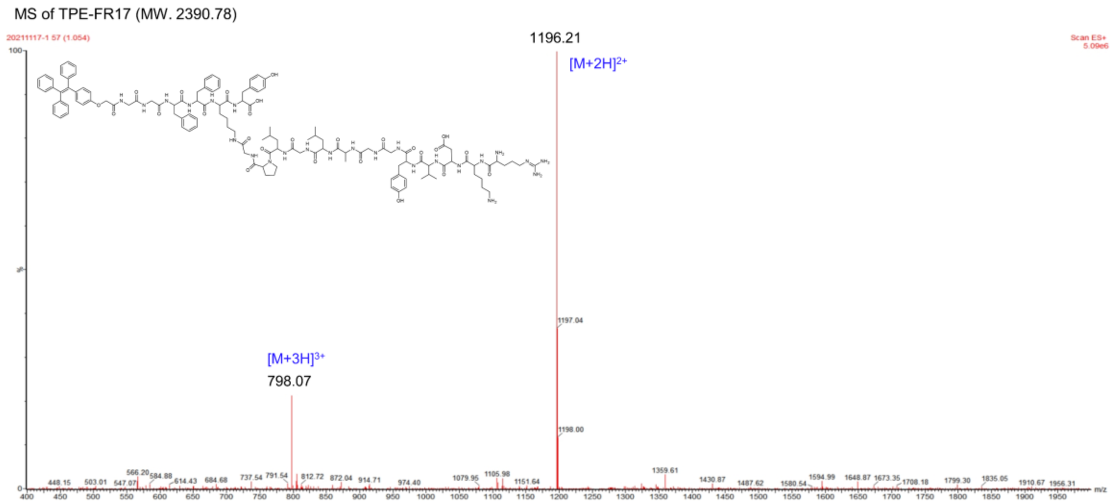

Supplementary Figure 28. Mass chromatogram of TPE-FR17 (MW. 2390.78).

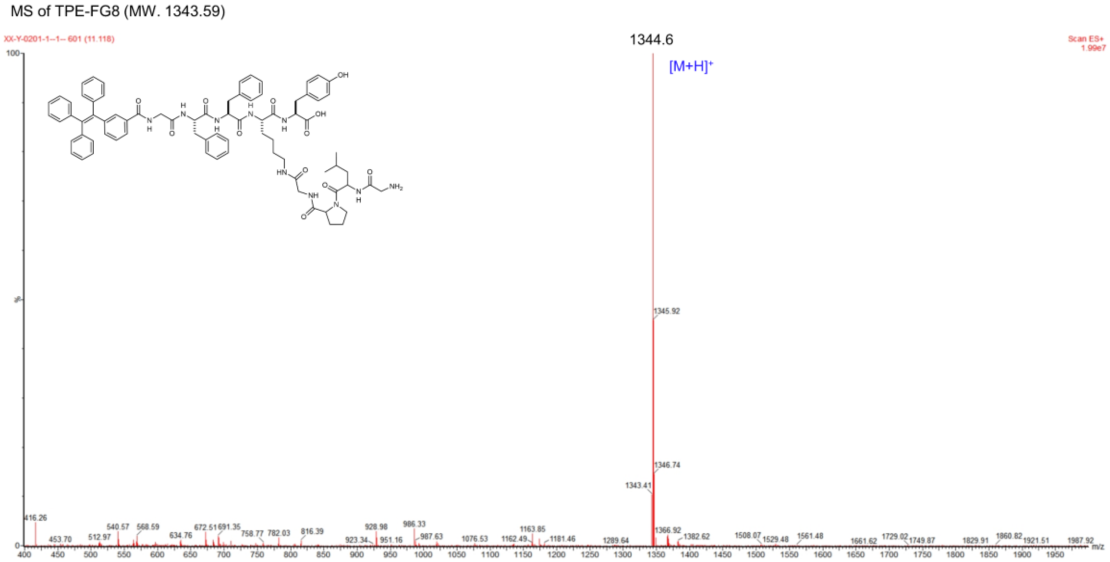

Supplementary Figure 29. Mass chromatogram of TPE-FG8 (MW. 1343.59).

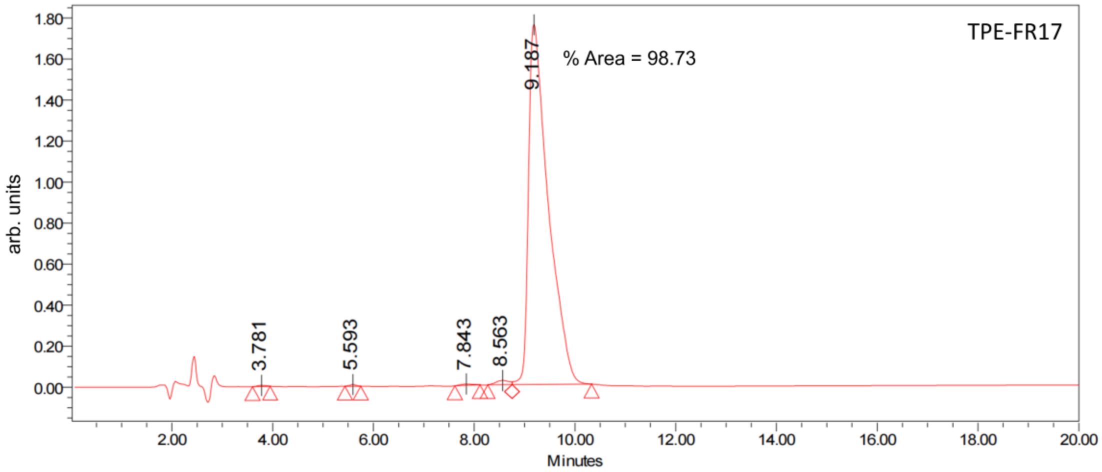

Supplementary Figure 30. HPLC of TPE-FR17 (%Purity = 98.73).

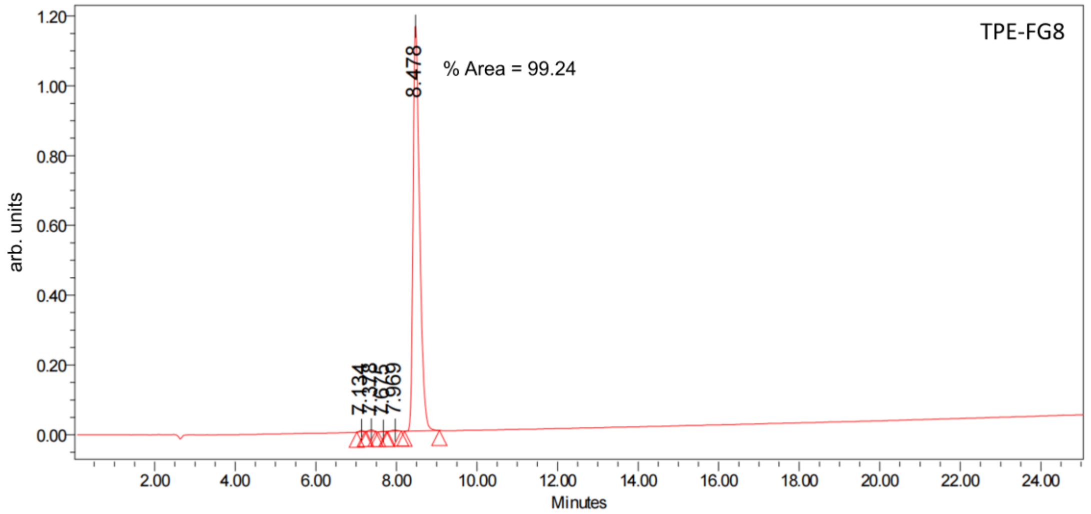

Supplementary Figure 31. HPLC of TPE-FG8 (%Purity = 99.24).

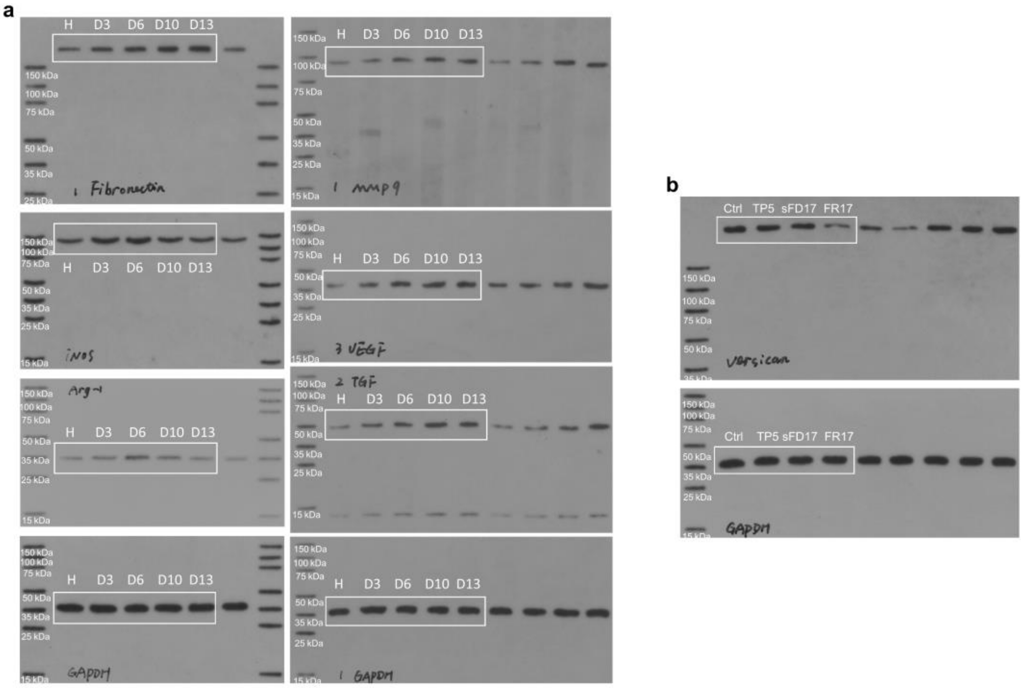

Supplementary Figure 32. The uncropped scans of all blots and gels in Supplementary Figure 10a and Supplementary Figure 15d.

Supplementary Table 1. The primer sequences for RT-qPCR analysis.

Target gene	F/R	Sequence (5’ → 3’)
M-Gapdh	F	GGTTGTCTCCTGCGACTTCA
	R	TGGTCCAGGGTTTCTTACTCC
M-Vegfa	F	GCTACTGCCGTCCGATTGAG
	R	ACTCCAGGGCTTCATCGTTACAG
M-Mmp9	F	CACAGCCAACTATGACCAGGAT
	R	CAGGAAGACGAAGGGGAAGA
M-Fn1	F	CTATTTACCAACCGCAGACTCAC
	R	TGCTTGTTTCCTTGCGACTT
M-Acta2	F	CAACTGGTATTGTGCTGGACTC
	R	ATCTCACGCTCGGCAGTAGT

References

1. Huang, D. W., Sherman, B. T. & Lempicki, R. A. Systematic and integrative analysis of large gene lists using DAVID bioinformatics resources. *Nat Protoc*. **4**, 44-57 (2009).
2. Villanueva, R. A. M. & Chen, Z. J. ggplot2: elegant graphics for data analysis, 2nd edition. *Meas. Interdiscip. Res.* **17**, 160–167 (2019).
3. Chen, H. & Boutros, P. C. VennDiagram: a package for the generation of highly-customizable Venn and Euler diagrams in R. *BMC Bioinformatics* **12**, 35 (2011).
