# Supplementary material for: Peptide nano-blanket impedes fibroblasts activation and subsequent formation of pre-metastatic niche
Source: Nat Commun. 2022 May 25;13:2906. doi: 10.1038/s41467-022-30634-8 (PMC9132894; doi:10.1038/s41467-022-30634-8)
Supplement: Supplementary file 3 — Reporting Summary [file 41467_2022_30634_MOESM3_ESM.pdf]

nature portfolio

Corresponding author(s): Min Han, Jianqing Gao

Last updated by author(s): Apr 28, 2022

Reporting Summary

Nature Portfolio wishes to improve the reproducibility of the work that we publish. This form provides structure for consistency and transparency in reporting. For further information on Nature Portfolio policies, see our Editorial Policies and the Editorial Policy Checklist.

Statistics

For all statistical analyses, confirm that the following items are present in the figure legend, table legend, main text, or Methods section.

n/a	Confirmed	
☐	☒	The exact sample size (n) for each experimental group/condition, given as a discrete number and unit of measurement
☐	☒	A statement on whether measurements were taken from distinct samples or whether the same sample was measured repeatedly
☐	☒	The statistical test(s) used AND whether they are one- or two-sided<br>*Only common tests should be described solely by name; describe more complex techniques in the Methods section.*
☐	☒	A description of all covariates tested
☐	☒	A description of any assumptions or corrections, such as tests of normality and adjustment for multiple comparisons
☐	☒	A full description of the statistical parameters including central tendency (e.g. means) or other basic estimates (e.g. regression coefficient) AND variation (e.g. standard deviation) or associated estimates of uncertainty (e.g. confidence intervals)
☐	☒	For null hypothesis testing, the test statistic (e.g. F, t, r) with confidence intervals, effect sizes, degrees of freedom and P value noted<br>*Give P values as exact values whenever suitable.*
☒	☐	For Bayesian analysis, information on the choice of priors and Markov chain Monte Carlo settings
☒	☐	For hierarchical and complex designs, identification of the appropriate level for tests and full reporting of outcomes
☐	☒	Estimates of effect sizes (e.g. Cohen's d, Pearson's r), indicating how they were calculated

Our web collection on statistics for biologists contains articles on many of the points above.

Software and code

Policy information about availability of computer code

Data collection	Data was collected using the software of the instrument described in each experiment. Confocal data was collected using Leica Application Suite X (LAS X) software. IVIS data was collected using Living Image software v4.3.1 (Perkin Elmer). Plate reader data was collected using BioTek Gen5 software. Flow cytometry data was collected using BD FACSDiva software v8.0.1 and analyzed using FlowJo software v10.6.2. Size distribution data was collected using Zetasizer software v7.13. Mass and tandem mass spectrometry were performed by Analyst TF v1.6 and PeakView software v1.2 (AB Sciex). Liquid chromatography-tandem mass spectrometry was auto-optimized by MassHunter Workstation Software vB.09.00, collected by 6400 Series Triple Quadrupole (Agilent Technologies). All-atom molecular dynamics simulation was constructed via AMBERTOOL, Gromacs 2018.4 package, VMD, PyMOL v1.8.2.2 and GRACE software. Bond lengths were constrained by the LINCS algorithms.
Data analysis	All statistical analysis were performed on Graphpad Prism 8.0.1. Flowcytometry data were analyzed on FlowJo software package (Flowjo V10). Chemical structures were drawn using Chemdraw v15.0. Images were analyzed by ImageJ v1.51j8. GO functional analysis was performed in the DAVID database. The Venn diagram was drawn using the VennDiagram package.

For manuscripts utilizing custom algorithms or software that are central to the research but not yet described in published literature, software must be made available to editors and reviewers. We strongly encourage code deposition in a community repository (e.g. GitHub). See the Nature Portfolio guidelines for submitting code & software for further information.

Data

Policy information about availability of data

All manuscripts must include a data availability statement. This statement should provide the following information, where applicable:

- Accession codes, unique identifiers, or web links for publicly available datasets
- A description of any restrictions on data availability
- For clinical datasets or third party data, please ensure that the statement adheres to our policy

The sorted CD11b+Ly6g+ MDSC RNAseq data are available on GEO (Accession: GSE181898). All the other data supporting the findings of this study are available within the article and its Supplementary Information files. A reporting summary for this article is available as a Supplementary Information files. Source data are provided with this paper.

Field-specific reporting

Please select the one below that is the best fit for your research. If you are not sure, read the appropriate sections before making your selection.

- [x] Life sciences
- [ ] Behavioural & social sciences
- [ ] Ecological, evolutionary & environmental sciences

For a reference copy of the document with all sections, see nature.com/documents/nr-reporting-summary-flat.pdf

Life sciences study design

All studies must disclose on these points even when the disclosure is negative.

Sample size	Sample sizes were based on previously published work of a similar nature (PMID: 31823615; PMID: 16341007), and chosen to meet the current standards for in vivo and in vitro experiments. Sample size for each experiment was set at least at 3 to obtain reliable results. Sample sizes were indicated in figures legends. Each sample represents independent biological replicates.
Data exclusions	No sample or data was excluded for analysis.
Replication	All experiments were repeated for at least three times and experimental findings were reproducible.
Randomization	The experimental groups were allocated randomly.
Blinding	The investigator was blinded to the group allocation during data collection. And blinding was performed for all analyses.

Reporting for specific materials, systems and methods

We require information from authors about some types of materials, experimental systems and methods used in many studies. Here, indicate whether each material, system or method listed is relevant to your study. If you are not sure if a list item applies to your research, read the appropriate section before selecting a response.

Materials & experimental systems

n/a	Involved in the study	
[ ]	[x]	Antibodies
[ ]	[x]	Eukaryotic cell lines
[x]	[ ]	Palaeontology and archaeology
[ ]	[x]	Animals and other organisms
[x]	[ ]	Human research participants
[x]	[ ]	Clinical data
[x]	[ ]	Dual use research of concern

Methods

n/a	Involved in the study	
[x]	[ ]	ChIP-seq
[ ]	[x]	Flow cytometry
[x]	[ ]	MRI-based neuroimaging

Antibodies

Antibodies used

The following primary antibodies and secondary antibody were used for western blotting.
Anti-Fibronectin (1:500, Cat. ab2413, Abcam, UK), RRID:AB_2262874,
Anti-MMP9 (1:1000, Cat. ab38898, Abcam, UK), RRID:AB_776512,
Anti-VEGFa (1:500, Cat. ab119, Abcam, UK), RRID:AB_298682,
Anti-TGF-β1 (1:1000, Cat. ab179695, Abcam, UK),
Anti-iNOS (1:1000, Cat. ab204017, Abcam, UK),
Anti-Arginase 1 (1:1000, Cat. ab124917, Abcam, UK), RRID:AB_10971357,
Anti-GAPDH (1:10000, Cat. ab181602, Abcam), RRID:AB_2630358,

Anti-Versican (1:1000, Cat. ab270445, Abcam, UK),
Anti-ANG2 (1:500, Cat. ab155106, Abcam, UK),
Anti-MMP2 (1:500, Cat. ab97779, Abcam, UK), RRID:AB_10696122,
Goat anti-Mouse IgG (H+L) (1:5000, Cat. 31160, Thermo Fisher), RRID:AB_228297,
Goat anti-Rabbit IgG (H+L) (1:5000, Cat. 31210, Thermo Fisher), RRID:AB_228334.

The following primary antibodies and secondary antibody were used for immunofluorescence.
Anti-VE-cadherin antibody (1:1000, Cat. Ab205336, Abcam, UK),
AF647 labeled goat anti-rabbit IgG (H+L) (1:100, Cat. 33113ES60, Yeasen, China),
Anti-αSMA (1:500, Cat. Ab7817, Abcam, UK), RRID:AB_262054
Anti-Vimentin (1:2000, Cat. Cy5134, Abways, China)
Anti-CD34 (1:500, Cat. Ab81289, Abcam, UK), RRID:AB_1640331,
Anti-MMP2 (1:200, Cat. 10373-2-ap, PTG, USA), RRID:AB_2250823,
Anti-MMP9 (1:1000, Cat. ab228402, Abcam, UK),
Anti-periostin (1:200, Cat. 19899-1-AP, PTG, USA), RRID:AB_10732682,
Anti-LOX (1:200, Cat. ab174316, Abcam, UK), RRID:AB_2630343,
Anti-Fibronectin (1:200, Cat. ab92572, Abcam, UK), RRID:AB_10561758,
Anti-CD11b (1:2000, Cat. ab133357, Abcam, UK), RRID:AB_2650514,
Anti-Gr-1 (1:200, Cat. ab25377, Abcam, UK), RRID:AB_470492,
Cy3 conjugated goat anti-rabbit IgG (1:500, Cat. 111-165-003, Jackson, USA), RRID:AB_2338000.
goat anti-rabbit IgG conjugated to HRP (1:2000, Cat. ab6721, Abcam, UK), RRID:AB_955447.

The following primary antibodies were used for flowcytometry and FACS.
FITC-antimouse-CD45 (1:500, Cat. 553079, BD, USA), RRID:AB_394609,
PE-antimouse-NK1.1 (1:500, Cat. 108708, Biolegend, USA), RRID:AB_313395,
PE-antimouse-CD3 (1:500, Cat. 100205, Biolegend, USA), RRID:AB_312662,
PE-antimouse-TER119 (1:500, Cat. 116207, Biolegend, USA), RRID:AB_313708,
PE-antimouse-CD19 (1:500, Cat. 152407, Biolegend, USA), RRID:AB_2629816,
APC-antimouse-CD11b (1:250, Cat. 101211, Biolegend, USA), RRID:AB_312794,
BV605-antimouse-MHC II (1:150, Cat. 107639, Biolegend, USA), RRID:AB_2565894,
BB700-CD11c (1:150, Cat. 566505, BD, USA), RRID:AB_2869773,
BV421-antimouse-Ly6c (1:100, Cat. 562727, BD, USA), RRID:AB_2737748,
PE/CF594-antimouse-Ly6g (1:150, Cat. 562700, BD, USA), RRID:AB_2737730,
BV711-antimouse-F4/80 (1:150, Cat. 123147, Biolegend, USA), RRID:AB_2564588,
PE/Cy7-antimouse-CD103 (1:100, Cat. 121426, Biolegend, USA), RRID:AB_2563691,
BV650-antimouse-CD206 (1:50, Cat. 141723, Biolegend, USA), RRID:AB_2562445,
PE/Cy7-antimouse-Ly6g (1:150, Cat. 127617, Biolegend, USA), RRID:AB_1877262.

For in vivo study, Antibody anti-PD1 (Cat. BE0146, Bio X Cell, USA, RRID:AB_10949053) was used.

Validation

All antibodies were verified by the supplier and each lot has been quality tested. All the antibodies used are from commercial sources and have been validated by the vendors. Validation data are available on the manufacturer's website.
For WB:
Anti-Fibronectin: rabbit, validated for ICC/IF, WB, IHC-P, reacts with mouse, human; https://www.abcam.com/fibronectin-antibody-ab2413.html
Anti-MMP9: rabbit, validated for WB, reacts with mouse, recombinant fragment; https://www.abcam.com/mmp9-antibody-ab38898.html
Anti-VEGFa: mouse, reacts with human, mouse, rat, pig; https://www.abcam.com/vegfa-antibody-vg76e-ab119.html
Anti-TGF-β1: rabbit, validated for WB, reacts with mouse, rat, human, recombinant fragment; https://www.abcam.com/tgf-beta-1-antibody-epr18163-ab179695.html
Anti-iNOS: Rabbit, validated for WB, reacts with mouse, rat, human; https://www.abcam.com/inos-antibody-ab204017.html
Anti-Arginase: Rabbit, validated for WB, reacts with mouse, human; https://www.abcam.com/liver-arginase-antibody-epr6671b-ab124917.html
Anti-GAPDH: Rabbit, validated for Flow Cyt (Intra), WB, IHC-P, ICC/IF, IP, reacts with mouse, rat, chicken, human, zebrafish, african green monkey, xenopus tropicalis; https://www.abcam.com/gapdh-antibody-epr16891-loading-control-ab181602.html
Anti-Versican: rabbit, validated for WB, IHC-P, reacts with mouse, human; https://www.abcam.com/versican-antibody-epr23374-151-ab270445.html
Anti-ANG2: rabbit, validated for WB, reacts with mouse, rat, human; https://www.abcam.com/angiopoietin-2ang2-antibody-epr28912-ab155106.html
Anti-MMP2: rabbit, validated for IHC-P, IP, ICC/IF, WB, reacts with human; https://www.abcam.com/mmp2-antibody-ab97779.html
Goat anti-Mouse IgG (H+L): goat, validated for WB, IHC, ICC/IF, Flow, IP, reacts with mouse; https://www.thermofisher.cn/cn/zh/antibody/product/Goat-anti-Mouse-IgG-H-L-Secondary-Antibody-Polyclonal/31160
Goat anti-Rabbit IgG (H+L): goat, validated for WB, IHC, ICC/IF, Flow, IP, reacts with rabbit. https://www.thermofisher.cn/cn/zh/antibody/product/Goat-anti-Rabbit-IgG-H-L-Secondary-Antibody-Polyclonal/31210

For immunofluorescence:
Anti-VE-cadherin: rabbit, validated for ICC/IF, WB, reacts with mouse; https://www.abcam.com/ve-cadherin-antibody-epr18229-ab205336.html
AF647 labeled goat anti-rabbit IgG (H+L): goat, has been tested for specific binding with Complete rabbit IgG by ELISA; https://www.yeasen.com/products/detail/369

Anti-αSMA: mouse, validated for ICC, IHC-P, WB, Flow Cyt, reacts with rat, human, predicted and reported to react with mouse, Sheep, Rabbit, Cow, Pig, Mammals, Baboon; https://www.abcam.com/alpha-smooth-muscle-actin-antibody-1a4-ab7817.html?productWallTab=ShowAll
Anti-Vimentin: rabbit, validated for WB, IHC-P, ICC/IF, Flow Cyt, reacts with mouse, rat, human; http://www.abways.com/showproduct.asp?cid=CY5134
Anti-CD34: rabbit, validated for WB, IHC-P, ICC/IF, IP, IHC-Fr, Flow Cyt (Intra), reacts with mouse, rat, human; https://www.abcam.com/cd34-antibody-ep373y-ab81289.html
Anti-MMP2: rabbit, validated for IF, IHC, IP, WB, ELISA, reacts with human, mouse, rat; https://www.ptglab.com/products/MMP2-Antibody-10373-2-AP.htm
Anti-MMP9: rabbit, validated for IP, IHC-P, IHC-Fr, WB, reacts with mouse, rat; https://www.abcam.com/mmp9-antibody-epr22140-154-ab228402.html
Anti-periostin: rabbit, validated for IF, IHC, ELISA, WB, reacts with human, mouse, rat; https://www.ptglab.com/products/POSTN-Antibody-19899-1-AP.htm
Anti-LOX: rabbit, validated for WB, IHC-P, ICC/IF, IP, Flow Cyt (Intra), reacts with mouse, rat, human; https://www.abcam.com/lox-antibody-epr4025-ab174316.html
Anti-Fibronectin: rabbit, validated for WB, IP, IHC-P, ICC, Flow Cyt (Intra), react swith mouse, rat, human; https://www.abcam.com/fibrinogen-alpha-chain-antibody-epr2919-ab92572.html
Anti-CD11b: rabbit, validated for WB, IHC-P, reacts with mouse, rat, human; https://www.abcam.com/cd11b-antibody-epr1344-ab133357.html
Anti-Gr-1: rat, validated for IHC-Fr, reacts with mouse; https://www.abcam.com/ly6g--ly6c-antibody-rb6-8c5-ab25377.html
Cy3 conjugated goat anti-rabbit IgG: mouse, validated for ICC/IF, WB, IP, ICC, IHC-Fr, reacts with human, mammals; https://www.jacksonimmuno.com/catalog/products/111-165-003
goat anti-rabbit IgG conjugated to HRP: goat, validated for IHC-P, WB, ELISA, Immunomicroscopy, Dot blot, ICC, IHC-Fr, reacts with rabbit. https://www.abcam.com/goat-rabbit-igg-hl-hrp-ab6721.html

For Flow Cytometry:
FITC-antimouse-CD45: rat, validated for Flow Cyt, reacts with mouse; https://www.bdbiosciences.com/en-us/products/reagents/flow-cytometry-reagents/research-reagents/single-color-antibodies-ruo/fitc-rat-anti-mouse-cd45.553079
PE-antimouse-NK1.1: mouse, validated for Flow Cyt, reacts with mouse; https://www.biolegend.com/en-us/products/pe-anti-mouse-nk-1-1-antibody-431
PE-antimouse-CD3: rat, validated for Flow Cyt, reacts with mouse; https://www.biolegend.com/en-us/products/pe-anti-mouse-cd3-antibody-47
PE-antimouse-TER119: rat, validated for Flow Cyt, reacts with mouse; https://www.biolegend.com/en-us/products/pe-anti-mouse-ter-119-erythroid-cells-antibody-1867
PE-antimouse-CD19: rat, validated for Flow Cyt, reacts with mouse; https://www.biolegend.com/en-us/products/pe-anti-mouse-cd19-antibody-13641.com)
APC-antimouse-CD11b: rat, validated for Flow Cyt, reacts with mouse, human; https://www.biolegend.com/en-us/products/apc-anti-mouse-human-cd11b-antibody-345
BV605-antimouse-MHC II: rat, validated for Flow Cyt, reacts with mouse; https://www.biolegend.com/en-us/products/brilliant-violet-605-anti-mouse-i-a-i-e-antibody-11988
BB700-CD11c: hamster, validated for Flow Cyt, reacts with mouse; https://www.bdbiosciences.com/en-us/products/reagents/flow-cytometry-reagents/research-reagents/single-color-antibodies-ruo/bb700-hamster-anti-mouse-cd11c.566505
BV421-antimouse-Ly6c: rat, validated for Flow Cyt, Immunofluorescence, reacts with mouse; https://www.bdbiosciences.com/en-us/products/reagents/flow-cytometry-reagents/research-reagents/single-color-antibodies-ruo/bv421-rat-anti-mouse-ly-6c.562727
PE/CF594-antimouse-Ly6g: rat, validated for Flow Cyt, reacts with mouse; https://www.bdbiosciences.com/en-us/products/reagents/flow-cytometry-reagents/research-reagents/single-color-antibodies-ruo/pe-cf594-rat-anti-mouse-ly-6g.562700
BV711-antimouse-F4/80: rat, validated for Flow Cyt, reacts with mouse; https://www.biolegend.com/en-us/products/brilliant-violet-711-anti-mouse-f4-80-antibody-10705
PE/Cy7-antimouse-CD103: Armenian Hamster, validated for Flow Cyt, reacts with mouse; https://www.biolegend.com/en-us/products/pe-cyanine7-anti-mouse-cd103-antibody-9899
BV650-antimouse-CD206: rat, validated for ICFC, Flow Cyt, reacts with mouse; https://www.biolegend.com/en-us/products/brilliant-violet-650-anti-mouse-cd206-mmr-antibody-8842
PE/Cy7-antimouse-Ly6g: rat, validated for Flow Cyt, reacts with mouse. https://www.biolegend.com/en-us/products/pe-cyanine7-anti-mouse-ly-6g-antibody-6139

For in vivo experiment:
Antibody anti-PD1: Syrian Hamster BKH cells transfected with mouse PD-1 cDNA, has been reported applicated for in vivo blocking of PD-1/PD-L signaling. https://bxcell.com/product/invivoplus-anti-m-pd-1/

Eukaryotic cell lines

Policy information about cell lines

Cell line source(s)

The B16F10 cells were purchased from the Cell Bank of Chinese Academy of sciences (Cat. TCM36, Shanghai, China) which was originally obtained from the American Type Culture Collection (Cat. CRL-6475, RRID:CVCL_0159, Manassas, USA). The mouse lung fibroblasts (MLF) were purchased from iCell Bioscience Inc. (Cat. iCell-0033a, Shanghai, China) which were originally isolated from mice pulmonary tissues and then transfected with SV40 through lentiviral. The bEnd3 cells were originally purchased from the Cell Bank of Chinese Academy of sciences (Cat. TCM40, Shanghai, China) which was originally obtained from the American Type Culture Collection (Cat. CRL-2299, RRID:CVCL_0170, Manassas, USA).

Authentication

Cell lines were not further authenticated after their receipt from the source.

Mycoplasma contamination	No mycoplasma contamination was found.
Commonly misidentified lines (See ICLAC register)	None of the cell lines used are listed in the ICLAC list.

Animals and other organisms

Policy information about studies involving animals; ARRIVE guidelines recommended for reporting animal research

Laboratory animals	C57BL/6 mice (male, 5-week-old) purchased from Slaccas (Shanghai, China) were adaptive fed for more than one week for subsequent experiments. The animals were maintained under standard laboratory housing conditions (25±1°C, 50% relative humidity and 12h/12h dark/light cycle where foods and water can be reached freely).
Wild animals	No wild animals were used in this study.
Field-collected samples	No field-collected samples were used in this study.
Ethics oversight	All the animal experiments (approval number: 21856) were conducted following the guidelines which have been approved by the Institutional Animal Care and Use Committee (IACUC) of Zhejiang University.

Note that full information on the approval of the study protocol must also be provided in the manuscript.

Flow Cytometry

Plots

Confirm that:

- [x] The axis labels state the marker and fluorochrome used (e.g. CD4-FITC).
- [x] The axis scales are clearly visible. Include numbers along axes only for bottom left plot of group (a 'group' is an analysis of identical markers).
- [x] All plots are contour plots with outliers or pseudocolor plots.
- [x] A numerical value for number of cells or percentage (with statistics) is provided.

Methodology

Sample preparation	For flow cytometry analysis, lung tissues harvested from mice were mechanically minced into 1-2 mm pieces using scissors and then dissociated into single cell suspension at 37 °C on a shaker for 30 min by enzymes. The digesting solution contains 2 mg/mL collagenase I (Cat. BS163, BioSharp, Germany), 2 mg/mL collagenase II (Cat. BS164, BioSharp, Germany) and DNase I (Cat. KGF008, KeyGEN BioTech., China). Digestion was stopped by adding 2 volumes PBS and filtered through a 70 μM cell strainer (Cat. CSS013070, Jet BIOFIL®, China). The cell suspension was centrifuged at 400 g for 5 min to discard the supernatant. Cell precipitations were then resuspended in 5 mL RBC lysis buffer (Cat. R1010, Solarbio, China) and centrifuged again to discard the supernatant. The single-cell-suspensions washed with PBS and resuspended were incubated with antibodies according to the manufacturer's protocols, and then analyzed by flow cytometry.
Instrument	BD Fortessa
Software	FlowJo software package (Flowjo V10)
Cell population abundance	In general, cells were first gated on FSC/SSC. Singlet cells were gated using FSC-H and FSC-A.
Gating strategy	The preliminary FSC/SSC gates of the starting cell population was set according to the size of single cells to discard cell debris. The staining cell populations were defined as positive in gathering compared to the unstained cell control. Further gating strategies were determined in preliminary experiments and presented in detail in supplementary information (Supplementary Figure 12 & Supplementary Figure 19).

- [x] Tick this box to confirm that a figure exemplifying the gating strategy is provided in the Supplementary Information.
